# Fusion Inhibition of Zika Virus Entry by a Teicoplanin Pseudoaglycone Derivative with Broad Antiviral Activity

**DOI:** 10.3390/pharmaceutics18070879

**Published:** 2026-07-17

**Authors:** Zoltán Kopasz, Ilona Bereczki, Krisztina Leiner, Henrietta Papp, Eszter Boglárka Lőrincz, Levente Sipos-Szabó, Kornélia Bodó, Eszter Szabó, Mónika Madai, Brigitta Zana, Réka Erdei, Gyula Batta, Tamás Kovács-Öller, Zoltán Varga, Dávid Bajusz, Gábor Kemenesi, Anikó Borbás, Anett Kuczmog

**Affiliations:** 1 National Laboratory of Virology, Szentágothai Research Centre, University of Pécs, Ifjúság útja 20, 7624 Pecs, Hungary; kopasz.zoltan@pte.hu (Z.K.);; 2 Faculty of Sciences, Institute of Biology, University of Pécs, Ifjúság útja 6, H-7624 Pecs, Hungary; 3 Department of Pharmaceutical Chemistry, University of Debrecen, Egyetem tér 1, H-4032 Debrecen, Hungary; 4 Doctoral School of Pharmaceutical Sciences, University of Debrecen, Egyetem tér 1, H-4032 Debrecen, Hungary; 5 Department of Organic Chemistry and Technology, Budapest University of Technology and Economics, 1111 Budapest, Hungary; 6 Medicinal Chemistry Research Group and Drug Innovation Centre, HUN-REN Research Centre for Natural Sciences, Magyar Tudósok krt. 2, H-1117 Budapest, Hungary; 7 Department of Organic Chemistry, University of Debrecen, H-4032 Debrecen, Hungary; 8 Doctoral School of Chemistry, University of Debrecen, Egyetem tér 1, H-4032 Debrecen, Hungary; 9 János Szentágothai Research Centre, University of Pécs, Ifjúság útja 20, 7624 Pecs, Hungary; 10 Biological Nanochemistry Research Group, Institute of Materials and Environmental Chemistry, HUN-REN Research Centre for Natural Sciences, Magyar Tudósok Körútja 2, H-1117 Budapest, Hungary; 11 Department of Physical Chemistry and Materials Science, Faculty of Chemical Technology and Biotechnology, Budapest University of Technology and Economics, Műegyetem rkp. 3., H-1111 Budapest, Hungary; 12 HUN-REN–UD Molecular Recognition and Interaction Research Group, University of Debrecen, H-4032 Debrecen, Hungary

**Keywords:** chikungunya virus, o’nyong-nyong virus, severe acute respiratory syndrome coronavirus 2, glycopeptide antibiotics, broad-spectrum antiviral, fusion inhibitor

## Abstract

**Background/Objectives**: The lack of effective antiviral therapies for many viral infections highlights the need for the development of new antiviral agents. The broad antiviral effects of glycopeptide antibiotics (GPAs) and their derivatives have been previously described. In our studies, we investigated the in vitro viral inhibitory activity of newly synthesized GPA derivatives against Zika virus (ZIKV), chikungunya virus (CHIKV), o’nyong-nyong virus (ONNV) and severe acute respiratory syndrome coronavirus 2 (SARS-CoV-2). **Methods**: Antiviral activity (EC50) and cytotoxicity (CC50) of the active compounds were determined using cell-based assays. The mechanism of action of the lead compound was investigated using binding and entry assays, cell-free virion pre-incubation, a virion destabilization assay, a liposome-based capsid protection assay, and molecular docking analysis. **Results**: Seven of the compounds were able to inhibit ZIKV and two compounds inhibited all four tested viruses. Among them, a teicoplanin pseudoaglycone derivative, compound **7**, showed the strongest antiviral activity, inhibiting all four viruses at low micromolar concentrations. Mechanistic studies demonstrated that compound **7** acts during an early stage of ZIKV infection and inhibits low-pH-triggered virus–liposome fusion. Molecular docking analysis suggested potential interactions between compound **7** and the viral envelope protein that could interfere with the conformational rearrangements required for membrane fusion. **Conclusions**: The present findings demonstrate that hydrophobic GPA derivatives, particularly compound **7**, exhibit promising broad-spectrum antiviral activity in vitro. Whether similar mechanisms contribute to the antiviral activity against other viruses remains unknown. The studied GPA derivatives are promising candidates for further pre-clinical and clinical development as broad-spectrum antivirals.

## 1. Introduction

Zika virus (ZIKV), a member of the Flaviviridae family, belongs to the genus Flavivirus, along with other significant pathogens such as yellow fever virus (YFV), West Nile virus (WNV), and dengue virus (DENV). These viruses are notorious for causing serious public health concern globally. ZIKV has been identified by the WHO as a research priority in the context of emergencies and ISIDORE has listed it as a “Priority Preparedness Pathogen” (WHO, ISIDORE). ZIKV has an approximately 11 kb positive-sense RNA genome. This RNA, when translated in the cytoplasm, forms a large polyprotein. This polyprotein is cleaved into three structural proteins: the capsid (C), pre-membrane/membrane (prM/M), and the envelope (E), along with seven non-structural proteins, namely NS1, NS2A, NS2B, NS3, NS4A, NS4B, and NS5. The virus primarily attaches to host cells via the E protein and enters through clathrin-mediated endocytosis [1]. The main vectors of ZIKV are mosquitoes, notably species from the *Aedes* genus, such as *Aedes aegypti* and *Aedes albopictus* [2]. ZIKV was first isolated from the serum of a rhesus monkey in Uganda in 1947 [3]. The first major mass outbreak was in 2007 on Yap Island Federated States of Micronesia [4]. From 2015 to 2016, the indigenous spread of ZIKV extended from Brazil to over 20 Latin American countries and reached North America [5,6,7]. In human infections, 80% are asymptomatic or present only mild symptoms, including fever, rash, muscle pain, and conjunctivitis [8]. On the other hand, ZIKV infection has been associated with significant neurological complications, including Guillain–Barré syndrome (GBS) and microcephaly in fetuses and newborns [9]. Since 2013, 31 countries have reported cases of microcephaly and central nervous system malformations associated with ZIKV infections. There are several vaccines under development, based on different generations of technologies, but none is close to being commercialized [10]. Currently, there is no specific treatment available against ZIKV. Chikungunya virus (CHIKV) and o’nyong-nyong virus (ONNV) belonging to the genus Alphavirus in the Togaviridae family are other emerging arthropod-borne viruses. The main vectors of CHIKV, like ZIKV, are *A. aegypti* and *A. albopictus*, while ONNV’s primary vectors are anopheline mosquitoes, *Anopheles funestus* and *Anopheles gambiae* [11,12]. CHIKV causes acute infection with high fever lasting 3–5 days followed by severe polyarthralgia [13]. In addition to acute disease, CHIKV infection may lead to chronic joint pain, severe organ dysfunction, encephalitis, particularly in elderly patients, and severe neonatal disease. Since 2023, an adult-only chikungunya vaccine has been available for prevention, but there is no specific antiviral drug against CHIKV. ONNV is known to be responsible for sporadic outbreaks in Africa; viral infection, similar to chikungunya fever, is associated with severe arthralgia [14]. No effective vaccine or drugs are available against ONNV. Besides developing vaccines to prevent serious viral diseases, antiviral drug development should also be a high priority to save lives. Testing potentially antiviral compounds is essential for the development of new medicines and is particularly relevant for viruses such as ZIKV, CHIKV and ONNV for which no specific drugs are available. Glycopeptide antibiotics (GPAs) are used to treat Gram-positive bacterial infections and have also been shown to have antiviral activity against different viruses including Ebola virus (EBOV), MERS-CoV, and SARS-CoV [15,16,17,18]. In addition, it was amply demonstrated that synthetically modified lipophilic derivatives of GPAs, obtained by deglycosylating glycopeptides and attaching hydrophobic groups to the resulting aglycones or pseudoaglycones, show a much more pronounced and much broader antiviral effect than natural antibiotics. Semisynthetic hydrophobic derivatives of the antibiotics vancomycin, teicoplanin, and ristocetin have been shown to inhibit influenza types A and B (IAV, IBV), both human immunodeficiency viruses 1 and 2 (HIV-1, HIV-2), hepatitis C (HCV), DENV, YFV, tick-borne encephalitis virus (TBEV), WNV, ZIKV, herpes simplex virus types 1 and 2 (HSV-1, HSV-2), respiratory syncytial virus (RSV), and various coronaviruses including human coronavirus (hCoV-229E), SARS-CoV, SARS-CoV-2, and MERS-CoV [16,18,19,20,21]. In addition to their ability to inhibit many different viruses, glycopeptide antibiotic derivatives act through different mechanisms, even within the same virus family. Among the inhibitory pathways described, the most common is that they act in the early stages of infection [16,18,20,22]. The teicoplanin analogue LCTA-949 has been reported to exert its inhibitory effect on the E protein of DENV in the early stages of infection. The E protein of flaviviruses is a promising target for inhibiting virus entry because it is essential for both the virus particle binding to host cell receptors and for the fusion of the viral membrane with the target cell membrane [20]. In the case of HCV, which also belongs to the Flavivirus family, derivatives of teicoplanin aglycone were observed to inhibit the replication mechanism [23]. Interestingly, alphaviruses have been overlooked in mapping the antiviral activity spectrum of glycopeptide derivatives. Furthermore, investigations against ZIKV have been remarkably sparse; the only existing data stems from our previous studies. These include the evaluation of two vancomycin hexapeptide derivatives—prepared via deglycosylation followed by Edman degradation and subsequent derivatization—and two teicoplanin pseudoaglycone perfluoroalkyl derivatives. Notably, in both cases, only one derivative from each pair exhibited activity against ZIKV [21,24]. In that latter study [24], we demonstrated that the attachment of perfluorobutyl and perfluorooctyl moieties to a teicoplanin pseudoaglycone core via a triazole-ethylene glycol/tetraethylene glycol linker yielded potent, non-toxic inhibitors against influenza, HSV, and hCoV. However, ZIKV has only been marginally investigated, and structural and mechanistic questions remain largely unanswered.

To address these limitations, the present study was designed to systematically explore the structural requirements for antiviral efficacy and to elucidate the underlying structure–activity relationships. Here, we report on the synthesis and antiviral evaluation of a series of hydrophobic glycopeptide derivatives based on a broader set of glycopeptide cores, extending the derivatization to the ristocetin aglycone scaffold. To evaluate the effect of the fluorinated chain length, perfluorobutyl, perfluorohexyl, and perfluorooctyl groups were employed as hydrophobic units. We also investigated whether the triazole ring and the ethylene glycol spacer are strictly required for the antiviral effect, and we determined their optimal lengths to identify the structural characteristics influencing antiviral activity. Furthermore, we extended the antiviral evaluations to a broader panel of emerging viruses, specifically targeting the flavivirus ZIKV and the alphaviruses CHIKV and ONNV, while also testing their effects against the coronavirus SARS-CoV-2 to map the breadth of the activity spectrum. Going beyond basic antiviral screening, we also aimed to unravel the underlying mechanism of action against ZIKV. The top-performing derivative, which exhibited the strongest inhibitory activity against this target virus alongside broad-spectrum antiviral efficacy against the other three pathogens, was selected for detailed mechanistic characterization. Time-dependent entry assays, binding assays, and computational modeling were used to determine whether these novel perfluoro alkylated glycopeptides block viral entry or interfere with later steps in the viral replication cycle.

## 2. Materials and Methods

### 2.1. General Information

**Scheme 1:**
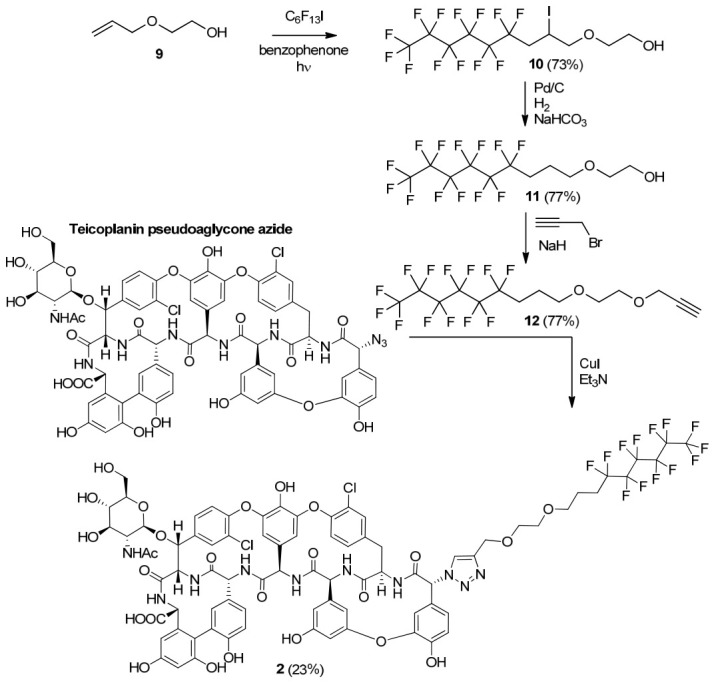
Synthesis of perfluorohexyl derivative **2** from teicoplanin pseudoaglycone azide by Cu(I)-catalyzed azide–alkyne click reaction.

**Scheme 2:**
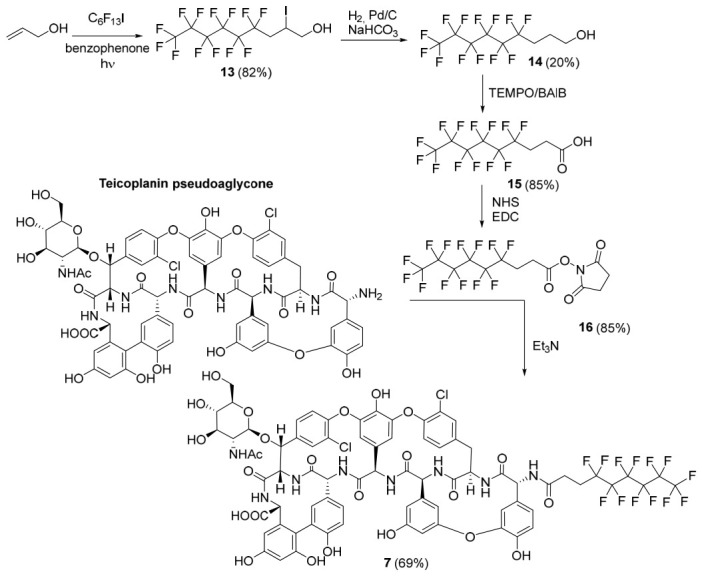
Synthesis of perfluorohexyl derivative **7** from teicoplanin pseudoaglycone via amide formation. TEMPO: tetramethylpiperidine 1 oxyl, BAIB: bis(acetoxy)iodobenzene, NHS: *N*-hydroxysuccinimide, EDC: 1-ethyl-3-(3-dimethylaminopropyl)carbodiimide.

**Scheme 3:**
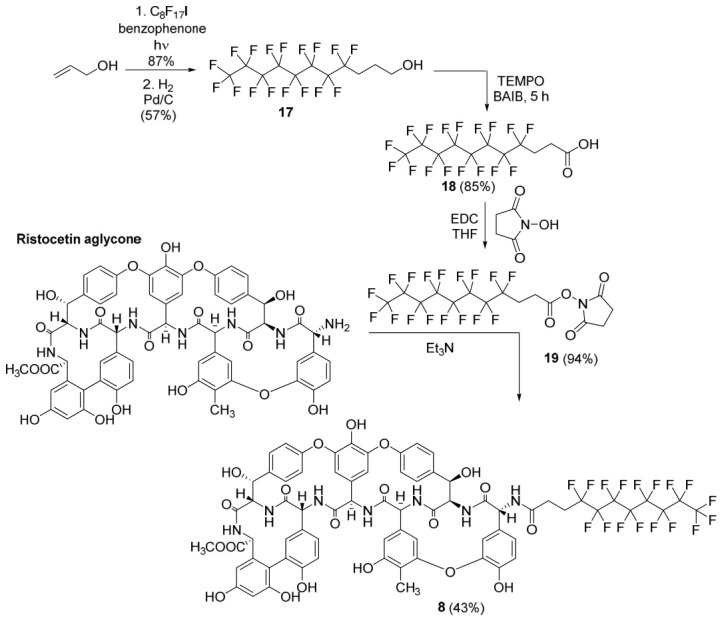
Synthesis of **8** from ristocetin aglycone via amide formation. TEMPO: tetramethylpiperidine 1 oxyl, BAIB: bis(acetoxy)iodobenzene, EDC: 1-Ethyl-3-(3-dimethylaminopropyl)carbodiimide, THF: tetrahydrofuran.

Teicoplanin was purchased from Beijing Mesochem Technology Co., Ltd. (Yizhuang, Beijing, China), vancomycin was purchased from BLD Pharmatech GmbH (Reinbek, Germany), allyl alcohol, perfluorohexyl iodide and perfluorooctyl iodide were purchased from Merck Life Science Kft., an affiliate of Merck KGaA (Darmstadt, Germany). The syntheses of teicoplanin pseudoaglycone, teicoplanin pseudoaglycone azide, ristocetin aglycone, perfluroalkyl teicoplanin derivatives **1**, **3–6** and compounds **9**, **14** and **19** were published previously [24,25,26,27,28]. Syntheses of teicoplanin derivatives **2** and **7** and ristocetin derivative **8** are shown in Scheme 1, Scheme 2 and Scheme 3. TLC was performed on a Kieselgel 60 F254 (Merck) with detection either by immersing into ammonium molybdate–sulfuric acid solution followed by heating or by using Pauly’s reagent for detection. Flash column chromatography was performed using Silica gel 60 (Merck 0.040–0.063 mm). The photoinitiated reactions were carried out in a borosilicate vessel by irradiation with a Hg-lamp giving maximum emission at 365 nm.

The ^1^H NMR (400, 500 and 700 MHz), ^13^C NMR (100, 125 and 176 MHz) and ^19^F (659 MHz, CPMG) spectra were recorded using Bruker’s (Berlin, Germany) DRX-400, Avance-II 500 and NEO-700 spectrometers at 298 K or 310 K in CDCl_3_ or DMSO-d_6_ solvent. Assignments were aided with 2D NMR spectra (^1^H-^1^H COSY, ROESY, ^1^H-^13^C HSQC, HSQC-TOCSY, HMBC, and ^1^H-^19^F HSQC) using the manufacturer’s Topspin program and pulse programs. Chemical shifts are referenced to Me_4_Si (0.00 ppm for ^1^H) or to solvent residual signals. NMR spectra and signal assignments of compounds **2**, **7** and **8** are as shown in the Supplementary Materials. MALDI-TOF MS measurements were carried out with a Bruker Autoflex Speed mass spectrometer equipped with a time-of-flight (TOF) mass analyzer. In all cases 19 kV (ion source voltage 1) and 16.65 kV (ion source voltage 2) were used. For reflectron mode, 21 kV and 9.55 kV were applied as reflector voltage 1 and 2, respectively. A solid phase laser (355 nm, ≥100 μJ/pulse) operating at 500 Hz was applied to produce laser desorption. 2,5-Dihydroxybenzoic acid (DHB) was used as a matrix and F_3_CCOONa as a cationizing agent in DMF. HRMS measurements were carried out on a maXis II UHR ESI-QTOF MS instrument (Bruker) in positive ionization mode. The following parameters were applied for the electrospray ion source: capillary voltage: 3.5 kV; end plate offset: 500 V; nebulizer pressure: 0.8 bar; dry gas temperature: 200 °C and dry gas flow rate: 4.5 L/min. Constant background correction was applied for each spectrum, the background was recorded before each sample by injecting the blank sample matrix (solvent). Na-formate calibrant was injected after each sample, which enabled internal calibration during data evaluation. Mass spectra were recorded by otofControl version 4.1 (build: 3.5, Bruker) and processed by Compass DataAnalysis version 4.4 (build: 200.55.2969). For analytical RP-HPLC a Waters 2695 Separations Module (Waters Corp., Milford, CT, USA) was used. The separation was carried out on a VDSpher PUR 100 C18-M-SE, 5 μm, 150 × 4.6 mm column at an injection volume of 10 μL, using a flow rate of 1.0 mL/min at 35 °C with a Waters 2996 DAD set at 225 nm and a Bruker MicroTOF-Q type Qq-TOF MS instrument (Bruker Daltonik, Bremen, Germany) as detectors. The following system was used for the elution: Solvent A: water + 0.1 *v*/*v*% TFA and Solvent B: MeCN. Gradient elution: from 0% of B to 90% from 0 to 45 min, 90% of B from 40 to 50 min and 0% of B from 51 to 56 min.

### 2.2. Compound ***10***

Allyloxyethanol **9** (1.02 mL, 10 mmol) and *n*-perfluorohexyl iodide (2.6 mL, 12 mmol, 1.2 equiv.) were dissolved in methanol (15 mL) and benzophenone (18 mg, 0.1 mmol, 0.01 equiv.) was added. Argon gas was bubbled through the solution for 10 min and the reaction mixture was irradiated with UV light for 20 min. The solvent was evaporated and the product was purified by flash column chromatography (hexane/acetone 8:2) to yield **10** (4.0 g, 73%) as a colorless syrup. *R*_f_ = 0.56 (hexane/acetone 7:3); ^1^HNMR (400 MHz, CDCl_3_): *δ* (ppm) 4.40 (p, 1H, *J* = 6.4 Hz, CI*H*), 3.83–3.59 (m, 6H, 3C*H*_2_), 3.12–2.94 (m, 1H, C*H*_2_), 2.84–2.65 (m, 1H, C*H*_2_), 2.23 (s, 1H, OH); ^13^C NMR (100 MHz, CDCl_3_): *δ* (ppm) from 120.4 to 108.1 highly split signs (6C, *C*F_2_ and *C*F_3_), 75.8, 72.4, 61.8 (3C, *C*H_2_), 37.9 (t, 1C, *C*H_2_), 15.0 (1C, *C*HI); HRMS: calculated for C_11_H_10_F_13_IO_2_Na^+^ 570.9410 [M+Na]^+^; found: 570.9401 *m*/*z*.

### 2.3. Compound ***11***

Compound **10** (4 g, 0.73 mol) was dissolved in methanol (25 mL) and argon gas was bubbled through the solution for 10 min. Palladium on activated charcoal (10%, 800 mg, 2.5 equiv.) and NaHCO_3_ (1.5 g, 1.82 mmol, 2.5 equiv.) were added. The reaction mixture was stirred overnight under H_2_ atmosphere, then filtered through celite, and the solvent was evaporated. The residue was dissolved in dichloromethane (100 mL) and the solution was washed with distilled water (2 × 20 mL), dried over anhydrous Na_2_SO_4_, filtered and the solvent was evaporated in vacuum. The product was purified by flash column chromatography (hexane/acetone 8:2) to yield 2.3 g (77%) **11** as a colorless syrup. *R*_f_ = 0.5 (hexane/acetone 7:3); ^1^H NMR (400 MHz, CDCl_3_) *δ* (ppm) 3.78–3.71 (m, 2H, C*H*_2_), 3.60–3.52 (m, 4H, 2C*H*_2_), 2.31–2.11 (m, 3H, C*H*_2_, O*H*), 1.96–1.86 (m, 2H, C*H*_2_); ^13^C NMR (100 MHz, CDCl_3_) *δ* (ppm) from 121.2 to 108.9 highly split signs (6C, *C*F_2_ and *C*F_3_), 72.2, 69.8, 61.9 (3C, *C*H_2_), 28.1 (t, 1C, *C*H_2_), 20.9 (1C, *C*H_2_); HRMS: calculated for C_11_H_11_F_13_O_2_Na^+^ 445.0444 [M+Na]^+^; found: 445.0433 *m*/*z*.

### 2.4. Compound ***12***

Compound **11** (1 g, 2.36 mmol) was dissolved in anhydrous THF and cooled in an ice-water bath. Then argon gas was bubbled through the solution for 10 min, then NaH (226 mg, 5.796 mmol, 60% dispersion in mineral oil) was added and the mixture was stirred for 30 min under argon atmosphere. After 30 min stirring, 1.2 mmol propargyl bromide (420 μL, 2.832 mmol, 1.2 equiv., 80% solution in toluene) was added and the reaction mixture was stirred overnight at room temperature. Then methanol (2 mL) and water (10 mL) were added to the mixture and it was stirred for 15–15 min. The solvent was evaporated, the residue was dissolved in dichloromethane (200 mL) and the solution was washed with distilled water (2 × 20 mL). The organic phase was dried over anhydrous Na_2_SO_4_, filtered and evaporated in vacuum. The product was purified by flash column chromatography (hexane/ethyl acetate 95:5) to yield **12** (838 mg, 77%) as a colorless syrup. *R*_f_ = 0.55 (hexane/ethyl acetate 9:1); ^1^H NMR (500 MHz, CDCl_3_) *δ* (ppm) 4.21 (d, 2H, *J* = 2.4 Hz, C*H*_2_ propargyl), 3.73–3.68 (m, 2H, C*H*_2_), 3.65–3.60 (m, 2H, C*H*_2_), 3.56 (t, 2H, *J* = 6.1 Hz, C*H*_2_), 2.43 (t, 1H, *J* = 2.4 Hz, C*H* propargyl), 2.27–2.13 (m, 2H, C*H*_2_), 1.94–1.85 (m, 2H, C*H*_2_); ^13^C NMR (100 MHz, CDCl_3_) *δ* (ppm) from 120.7 to 108.8 highly split signals (6C, *C*F_2_ and *C*F_3_),79.6 (1C, C_q_ propargyl), 74.6 (1C, *C*H propargyl), 70.2, 69.9, 69.2 (3C, *C*H_2_), 58.5 (1C, *C*H_2_ propargyl) 28.1 (t, 1C, *C*H_2_), 20.9 (1C, *C*H_2_); HRMS: calculated for C_14_H_13_F_13_O_2_Na^+^ 483.0600 [M+Na]^+^; found: 483.0595 *m*/*z*.

### 2.5. Compound ***2***

Teicoplanin pseudoaglycone (TC) azide (143 mg, 0.1 mmol) and compound **12** (55 mg, 0.12 mmol, 1.2 equiv.) were dissolved in anhydrous dimethylformamide (5 mL). Triethylamine (2 equiv., 28 μL, 0.2 mmol) and Cu(I)-iodide (4 mg, 0.02 mmol) were added, and the reaction mixture was stirred under argon atmosphere for 2 days. Then Na_2_S (5 mg, 0.064 mmol) dissolved in 1 mL of water was added. After 10 min of stirring the solvent was evaporated in vacuum (butanol was added to prevent foaming) and the residue was purified by flash column chromatography (acetonitrile/water 95:5→93:7) to yield compound **2** (44 mg, 23%) as a yellowish solid foam. *R*_f_ = 0.33 (acetonitrile/water 9:1); HRMS: calculated for C_80_H_68_Cl_2_F_13_N_10_O_25_Na_2_^+^: 1931.3316 [M-H+2Na]^+^; found: 1931.3324 *m*/*z*. NMR characterization can be found in the Supplementary Materials.

### 2.6. Compound ***15***

Compound **14** (293 mg, 0.775 mmol) was dissolved in dichloromethane (5 mL) and distilled water (2.5 mL) was added. 2,2,6,6-Tetramethylpiperidine 1-oxyl (TEMPO, 20 mg, 0.139 mmol, 0.18 equiv.) and (diacetoxyiodo)benzene (BAIB, 749 mg, 2.325 mmol, 3 equiv.) were added and the reaction mixture was stirred for 5 h. Then it was neutralized with 10% aqueous solution of Na_2_S_2_O_3_ (3 mL), diluted with dichloromethane (100 mL), shaken, and then separated. The aqueous phase was extracted with dichloromethane (2 × 100 mL), and the combined organic phase was dried over Na_2_SO_4_, filtered and the solvent was evaporated. The product was purified by flash column chromatography (hexane/acetone 8:2) to yield compound **15** (259 mg, 85%) as a white powder. *R*_f_ = 0.4 (hexane/acetone 7:3); ^13^C NMR (100 MHz, methanol-d_4_): *δ* (ppm) 175.5 (1C, C=O), 119.7, from 120.0 to 109.8 highly split signals (6C, *C*F_2_ and *C*F_3_), 27.5 (t, 1C, *C*H_2_), 26.0 (1C, *C*H_2_).

### 2.7. Compound ***16***

Compound **15** (200 mg, 0.51 mmol) was dissolved in the mixture of anhydrous dichloromethane (5 mL) and anhydrous THF (5 mL) and under argon atmosphere N-hydroxysuccinimide (65 mg, 0.561 mmol, 1.1 equiv.) was added and the mixture was cooled to 0 °C. After 10 min, 1-ethyl-3-(3-dimethylaminopropyl)carbodiimide hydrochloride (EDC, 108 mg, 0.561 mmol, 1.1 equiv.) was added and the reaction mixture was stirred overnight. Then the solvent was evaporated and the product was purified by flash column chromatography (hexane/acetone 8:2) to yield **16** (224 mg, 90%) as a white powder.

*R*_f_ = 0.32 (hexane/acetone 7:3); ^1^H NMR (400 MHz, CDCl_3_): *δ* (ppm) 3.02–2.94 (m, 2H, C*H*_2_), 2.86 (s, 4H, succinimide C*H*_2_), 2.71–2.50 (m, 2H, C*H*_2_); ^13^C NMR (100 MHz, CDCl_3_): *δ* (ppm) 168.9 (2C, succinimide *C*=O), 166.9 (1C, *C*=O), from 120.0 to 111.1 highly split signals (6C, *C*F_2_ and *C*F_3_), 26.4 (t, 1C, *C*H_2_), 25.7 (2C, succinimide *C*H_2_), 23.0 (1C, C*H*_2_).

### 2.8. Compound ***7***

Teicoplanin pseudoaglycone (TC) (136 mg, 0.1 mmol) was dissolved in anhydrous dimethylformamide (3 mL) and triethylamine (21 μL, 0.15 mmol, 1.5 equiv.) was added. Under argon atmosphere compound **16** (62 mg, 0.13 mmol, 1.3 equiv.) was added, and the reaction mixture was stirred overnight. Then the solvent was evaporated and the product was purified by flash column chromatography to yield compound **7** (119 mg, 69%) as a dirty white solid. *R*_f_ = 0.2 (toluene/methanol 1:1 containing 1.0 *v*/*v*% acetic acid); HPLC purity 100%, retention time: 29.66 min; MALDI-TOF-MS: calculated for C_75_H_61_Cl_2_F_13_N_8_O_24_ 1797.286 [M+Na]^+^; found 1797.271; NMR characterization can be found in the Supplementary Materials.

### 2.9. Compound ***8***

Ristocetin aglycone (100 mg, 0.085 mmol) was dissolved in anhydrous dimethylformamide (3 mL) and triethylamine (18 μL, 0.13 mmol, 1.5 equiv.) was added. Under argon atmosphere compound **19** (65 mg, 0.11 mmol, 1.3 equiv.) was added, and the reaction mixture was stirred overnight. Then the solvent was evaporated and the product was purified by flash column chromatography to yield compound **8** (60 mg, 43%) as a dirty white solid. *R*_f_ = 0.69 (toluene/methanol 1:1 containing 1.0 *v*/*v*% acetic acid); MALDI-TOF-MS: calculated for C_71_H_54_F_17_N_7_O_20_ 1670.304 [M+Na]^+^; found 1670.408; NMR characterization can be found in the Supplementary Materials.

### 2.10. Virus and Cell Lines

The Vero E6 cell line, derived from the kidney epithelial cells of the African green monkey (*Cercopithecus aethiops*) (ATCC^®^ CRL-1586^TM^), and A549, a human lung adenocarcinoma epithelial cell line (ATCC, CCL-185), were used in our in vitro antiviral screens. The cells were maintained at 37 °C under 5% CO_2_ in Dulbecco’s modified Eagle medium (DMEM, Lonza, Basel, Switzerland), supplemented with 10% fetal bovine serum (FBS, Biosera, France) and 1% penicillin/streptomycin antibiotics (PS, Lonza, Switzerland). The following viruses were used: ZIKV (MR766, Uganda), SARS-CoV-2 (B.1.5 (G) isolate, Hungarian), CHIKV (Guatemala), ONNV (UVE/ONNV/UNK/SN/Dakar 234). All virus stocks were propagated on Vero E6 cells and stored at −80 °C. Upon infection cell culture media were replaced with media containing 2% FBS and 1% PS.

### 2.11. Cytotoxicity Assay

To measure the cytotoxicity effect of compounds and determine the 50% cytotoxic concentration (CC_50_), the 3-[4,5-dimethylthiazol-2-yl]-2,5-diphenyltetrazolium bromide (MTT) colorimetric cell viability assay (MTT Cell Proliferation Assay, Roche, Grenzach-Wyhlen, Germany) was performed. To determine the minimal cytotoxicity concentration, the MCC (compound concentration producing minimal changes in cell morphology), we used microscopic observation by an inverted Nikon ECLIPSE Ti-U Serieslipse fluorescence microscope (Nikon, Tokyo, Japan). Compounds were tested over a concentration range of 0–400 μM for the initial cytotoxicity assessment.

### 2.12. Plaque Assay

To determine the infectious viral titer of the virus, a stock plaque assay was used. Cells were grown in a 24-well plate at a density of 3 × 10^5^ for one day (37 °C, 5% CO_2_) before the infection. We prepared a tenfold serial dilution of the virus stock solutions. After one hour of infection, the wells were covered with carboxymethyl cellulose to achieve a final concentration of 1%, and the plates were incubated for five days. Following fixation with paraformaldehyde, the cells were stained with 0.25% crystal violet and plaques were counted. The infectious viral titer was then calculated with the following formula: pfu/mL = avg. plaque number × (1/dilution) × (1000/infection volume (µL)).

### 2.13. CPE Inhibition Assay

In the context of ZIKV, 1 × 10^4^ Vero E6 cells per well were seeded in a 96-well tissue culture plate and incubated (37 °C, 5% CO_2_) overnight. For SARS-CoV-2, CHIKV, and ONNV 3.5 × 10^4^ Vero E6 cells were seeded the day before the infection. To measure the inhibitory effect of compounds, following a microscopic observation, the 3-[4,5-dimethylthiazol-2-yl]-2,5-diphenyltetrazolium bromide (MTT) colorimetric cell viability assay (MTT Cell Proliferation Assay, Roche, Germany) was employed. The cells were treated with the compounds diluted at indicated concentrations (from 0.3 µM to 60 µM) for the entire duration of the experiment. The control group was treated with an equal concentration of DMSO. After two hours of ZIKV infection at a multiplicity of infection (MOI) of 1, the supernatant was discarded, and the cells were incubated for five days with the compounds. SARS-CoV-2 and CHIKV infections were carried out with MOI of 0.1 for half an hour, while ONNV for 1 h with MOI of 0.1, and incubated for 3 days. Following the appropriate incubation time and microscopic examination, our measurements were carried out using 50 µL MTT working solution per 100 µL sample. The absorbance was measured at 560 nm with Crocodile 5in1 Mini Workstation (Berthold, Bad Wildbad, Germany). Three biological replicates of each concentration were used. Three biological replicates of the positive and negative controls were used.

### 2.14. Time of Drug Addition Assay

Vero E6 cells were seeded on 96-well plates at 3.5 × 10^4^ well and incubated (37 °C, 5% CO_2_) overnight. The cells were infected for 1 h with MOI of 1. Cells were treated with 50 µM of compound **7**. Cells were treated at three different times: 1 h before infection, concurrently with the infection, and one hour post-infection. After the infection, the supernatant was discarded and the cells were incubated for 24 h with the media, then RNA was isolated from the supernatant and quantified using qRT-PCR (see below).

### 2.15. Entry and Binding Assay

Vero E6 cells were seeded on 96 well plate 3.5 × 10^4^ well and incubated (37 °C, 5% CO_2_) overnight. 1 h before the infection we put the cells at 4 °C. During the entry assay cells were infected for 1 h at 4 °C and MOI of 1, then washed twice with PBS. Afterward, they were treated with 50 µM of compound **7**, lasting for 1 h (37 °C, 5% CO_2_). During the binding assay the cells were infected as before for 1 h at 4 °C and at the same time treated with 50 µM of compound **7**. Following both of the treatments, the cells were washed once with PBS, then RNA was isolated from the cells. Viral RNA was quantified using qRT-PCR (see below). This assay was developed based on the methods established by Lu et al. [29].

### 2.16. RNA Isolation and Quantitative Reverse Transcription PCR (qRT-PCR)

For viral RNA isolation, the Total RNA Miniprep Kit (New England Biolabs, Ipswich, USA) was used, following the manufacturer’s instruction. After RNA elution, samples were stored at −80 °C until use. For the determination of viral RNA quantity, we used the Luna^®^ Universal Probe One-Step RT-qPCR Kit (New England Biolabs, Ipswich, MA, USA). The primers used were ZIKV_NS1_F: AAA AGG AAA CGA GAG ATG TGG CA, ZIKV_NS1_R: CAT TCT CCT CTA GGA TAG CAT, and the probe was FAM-5′-CCC GCA GAT-3′ /ZEN/ 5′-TGG CAG CAG-3′-BHQ1. The reaction was performed as follows: 1 cycle at 55 °C for 10 min, 1 cycle at 95 °C for 1 min, followed by 40 cycles at 95 °C for 10 s and 60 °C for 30 s, using the CFX Opus Dx Real-Time PCR Systems (Bio-Rad, San Francisco, CA, USA). To determine the copy number, we prepared a standard curve. This involved initially purifying viral cDNA (DNA Gel Extraction Kit, New England Biolabs, USA) using gel electrophoresis, followed by determining the DNA concentration with the aid of a Qubit dsDNA BR Assay kit (ThermoFisher, Invitrogen, Waltham, MA, USA) based on the manufacturer’s protocol. Then we made a series of tenfold dilutions, which were included alongside each PCR reaction.

### 2.17. Inactivation Assay

We investigated whether the anti-ZIKV effects of compound **7** interact with the virus itself, in a cell-free environment. The virus stock (apx. 5,000,000 PFU/mL) was incubated in solutions of 50 µM compound **7** for 2 h at 37 °C. Then it was diluted 100-fold to eliminate the compounds from the sample and plaque assays were performed as previously described.

### 2.18. Virion Destabilization Assay

Undiluted 50 µL virus stock solution was incubated in the presence or absence of 50 µM of compound **7** for 1 h. After treatment, 80 µg/mL RNase A (Sigma-Aldrich, St. Louis, MO, USA) was added. One hour later Proteinase K (New England Biolabs, Ipswich, MA, USA) was added at 1 mg/mL for a 1 h incubation to inactivate the RNase. Then we isolated the viral RNA and performed qRT-PCR (see above). As a positive control we treated our stock solution with 20% ethanol. The optimization is based on the following work [29].

### 2.19. Molecular Modeling

Briefly, protein structures were prepared with the Protein Preparation Wizard of the Schrödinger suite with default settings [30], and prospective binding sites were mapped with the FTMap webserver [31,32]. The structure of compound **7** was prepared with Schrödinger Ligprep [33], conformationally sampled with Macromodel and docked to the respective binding sites by either Glide SP [34,35] or the Induced Fit Docking protocol of the Schrödinger suite [36], and the binding poses were rescored with the Prime MM/GBSA protocol [37]. For a fully detailed description of the respective modeling protocols, please see Supplementary Note S1 [38,39,40,41,42,43,44]. Except for the FTMap webserver, all software packages used were part of Schrödinger Release 2024-2.

### 2.20. Immunofluorescence Assay (IFA)

We seeded 9 × 10^4^ Vero E6 cells per well in an 8-well glass chamber slide (Biologix, Cat# 07-2108). After treatment and infection (see previous methods) cells were fixed with ice-cold methanol for 30 min. Then, 1% BSA (ThermoFisher, Waltham, MA, USA) in phosphate-buffered saline (PBS) solution was used for blocking. Subsequently, the cells were stained using a primary Zika virus NS1 Monoclonal Antibody (Invitrogen, Cat# MA5-24585, 1:100) for 1 h at 37 °C and secondary Goat Anti-Mouse IgG H&L Alexa Fluor^®^ 488 (Abcam, Cambridge, UK, Cat# ab150113, 1:1000) for 30 min at 37 °C. Finally, the cells were stained with 4′,6-diamidino-2-phenylindole (DAPI) solution (ThermoFisher, Invitrogen, Waltham, MA, USA, Cat# D1306) at room temperature for 10 min. Between each step, the cells were washed five times with PBS. Images of the cells were captured using a Zeiss LSM 710 confocal microscope (Plan Apochromat 10×, 20×, and 63× objectives (NA: 0.45, 0.8, 1.4, Carl Zeiss Inc., Jena, Germany)) with normalized laser power and filter settings with open pinhole in low magnification or by making 0.5 μm thin optical sections.

### 2.21. Capsid Protection Assay to Monitor Fusion of Virions with Liposomes

The liposome composition was designed to mimic late endosomal membranes, as previously described [45,46,47,48]. Liposomes were made with 1,2-dioleoylsn-glycero-3-phosphocholine (DOPC) (Avanti Polar Lipids), 1,2-dioleoyl-sn-glycero-3-phosphoethanolamine (DOPE) (Avanti Polar Lipids), soy (PI), bis(monoacylglycero)phosphate, S,R isomer (BMP), and cholesterol (Avanti Polar Lipids) at a 4/1/1/2/4 molar ratio in TAN buffer (20 mM triethanolamine, 100 mM NaCl, pH 8.0). Following five freeze/thaw cycles, liposomes were prepared by extrusion through a polycarbonate filter with 0.2 μm pore size (Nuclepore, Whatman Inc., Kent, UK), using a LIPEX extruder (Northern Lipids Inc., Burnaby, BC, Canada). For trypsin-containing liposomes, 3.8 mg of trypsin was added to 0.38 mL of lipids (2.8 mg each) after the fifth freeze/thaw cycle, prior to extrusion. Liposomes were separated from unincorporated trypsin by size-exclusion chromatography using a qEVsingle column with an automatic fraction collector (AFC, IZON Science Ltd., Christchurch, New Zealand). The virus was incubated with various concentrations of compounds (0, 10, 50 μM) for 45 min at 37 °C in TAN buffer (pH 8.0) prior to addition of 20 μL of liposomes for 5 min. After incubation with liposomes, 2.5 μL of 1 M sodium acetate (pH 5.0) was added to drop the pH to 5.3 for 5 min. Samples were neutralized by adding 2.5 μL of 2 M triethanolamine (pH 8.1) and incubated at 37 °C for 20 min to allow trypsin digestion. Laemmli sample buffer with 2-mercaptoethanol was added, and samples were boiled for 20 min before separation by 4–20% Mini-PROTEAN^®^ TGX™ Precast Protein Gels (Bio-Rad). Proteins were transferred to a nitrocellulose membrane, 0.45 µm, using a semidry transfer apparatus. The following antibodies were used for detection of envelope and capsid protein, respectively: ZIKV capsid protein antibodies (1:800) (GeneTex, GTX133317, Hsinchu, Taiwan), ZIKV envelope protein antibodies (1:1000) (GeneTex, GTX133314, Hsinchu, Taiwan). The membrane was developed with enhanced Clarity™ Western ECL Substrate (Bio-Rad, San Francisco, CA, USA), and chemiluminescent signals were captured using an Azure 200 Imaging System (Azure Biosystems, Dublin, CA, USA).

### 2.22. Phospholipidosis Assay

Vero E6 cells were seeded in 96-well plates and treated with compound **7** at the indicated concentrations for 48 h. Propranolol (30 µM) was used as a positive control and DMSO as vehicle control. Phospholipid accumulation was detected using LipidTOX™ Deep Red phospholipidosis stain (Thermo Fisher Scientific, Waltham, MA, USA, H34158) according to the manufacturer’s instructions. Nuclei were counterstained with Hoechst 33342. Representative images were acquired by confocal microscopy.

### 2.23. Statistical Analysis

For the statistical analysis, we used version 9.5 of GraphPad Prism (GraphPad Software, Boston, MA, USA). To determine the EC_50_ in the CPE inhibition assay, we employed non-linear regression analysis. qRT-PCR results were analyzed using a Student *t*-test. qPCR data represent the mean and standard error of the mean (±SEM) and *p* < 0.05 was marked as statistically significant.

## 3. Results

### 3.1. Synthesis of Compounds

**Figure 1:**
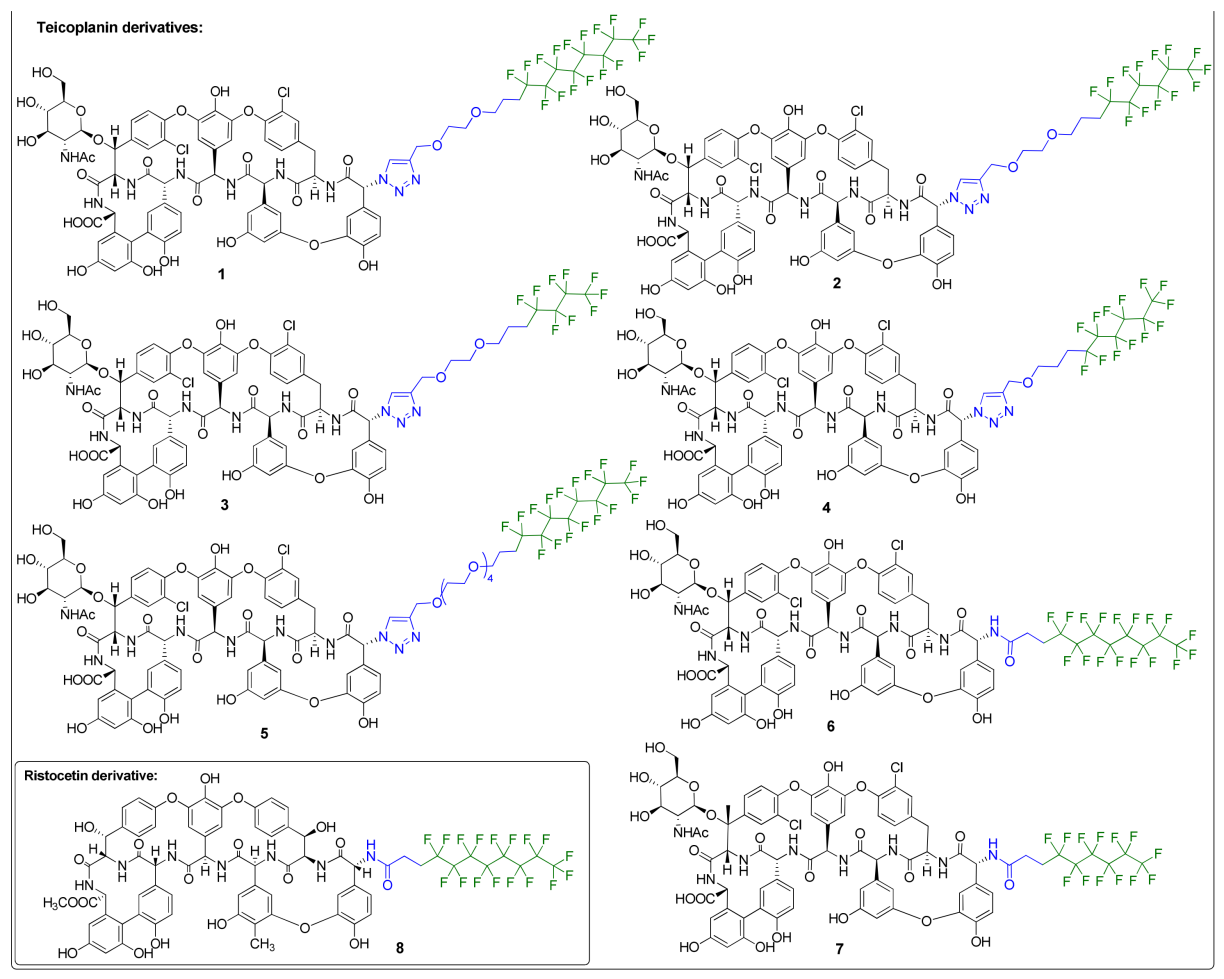
Structures of perfluoroalkyl derivatives of teicoplanin pseudoaglycone (**1–7**) and ristocetin aglycone (**8**).

Hydrophobic glycopeptide antibiotic aglycone/pseudoaglycone derivatives were prepared by attaching perfluoroalkyl groups of various sizes to the *N*-terminal amino acid unit of the heptapeptide scaffolds derived from native antibiotics (Figure 1). Partial deglycosylation was performed for teicoplanin and complete deglycosylation for ristocetin before hydrophobic modification, yielding a series of eight compounds, including seven teicoplanin pseudoaglycone derivatives (**1–7**), and one ristocetin aglycone derivative (**8**). We chose perfluoroalkyl groups of various lengths (C_4_–C_8_) as hydrophobic units because they do not have a membrane perturbing effect [49] and therefore are not expected to make the antibiotic derivatives cytotoxic [24]. In addition to varying the perfluoroalkyl chain length, we aimed to investigate the influence of the linker between the glycopeptide scaffold and the perfluoroalkyl moiety on the antiviral activity. Consequently, derivatives were synthesized using linkers of diverse lengths and chemical natures. The *N*-terminus of the glycopeptide antibiotics was utilized for the formation of amide bonds (**6–8**); alternatively, an azide group was introduced at the *N*-terminus to facilitate connection via a triazole ring through alkyne–azide “click” chemistry (**1–5**). In the case of the triazole-linked derivatives, the impact of further linker elongation was explored by incorporating shorter ethylene glycol (**1–3**) and longer tetraethylene glycol spacers (**5**). This series of compounds allows us to study the impact of the peptide backbone, the size of the perfluoro group and the linker region on the antiviral activity.

The synthesis of compounds **1**, **3–6** was previously reported, while **2**, **7** and **8** are new compounds, designed and produced for this study.

Commercially available perfluorohexyl iodide was conjugated to olefin **9** by a light-induced radical addition reaction [50], and the resulting **10** was deiodinated by catalytic hydrogenation to give **11** (Scheme 1). Next, fluorinated alcohol **11** was converted to propargyl ether **12** by alkylation with propargyl bromide. Finally, a copper(I)-catalyzed 1,3-dipolar cycloaddition reaction of **12** with teicoplanin pseudoaglycone azide [27] provided the perfluorohexyl teicoplanin derivative **2** having a triazolyl ethyleneglycol linker region.

For the synthesis of the perfluorohexyl teicoplanin derivative **7**, the fluorous carboxylic acid **15** was prepared from allyl alcohol and perfluorohexyl iodide in three steps including photoinitiated addition, catalytic hydrogenolysis and oxidation with the TEMPO–BAIB reagent combination (Scheme 2). Compound **15** was converted to the active ester **16** with *N*-hydroxysuccinimide in the presence of EDC. Finally, by acylating the *N*-terminal amino group of the teicoplanin pseudoaglycone with the active ester **16**, compound **7** was obtained, in which the perfluorohexyl group is directly connected to the glycopeptide skeleton.

Ristocetin aglycone derivative **8** containing a perfluorooctyl substituent was prepared in the same way as the synthesis of **7** but using perfluorooctyl iodide as the fluorous reactant (Scheme 3). Active ester **19**, prepared from perfluorooctyl iodide and allyl alcohol in four steps, was conjugated to ristocetin aglycone [26] by amide coupling to provide compound **8** bearing the perfluorous group directly connected to the peptide skeleton. This compound is the ristocetin counterpart of teicoplanin derivative **6** containing exactly the same hydrophobic group.

### 3.2. GPAs Show Antiviral Activities Against Different Emerging Viruses

**Figure 2:**
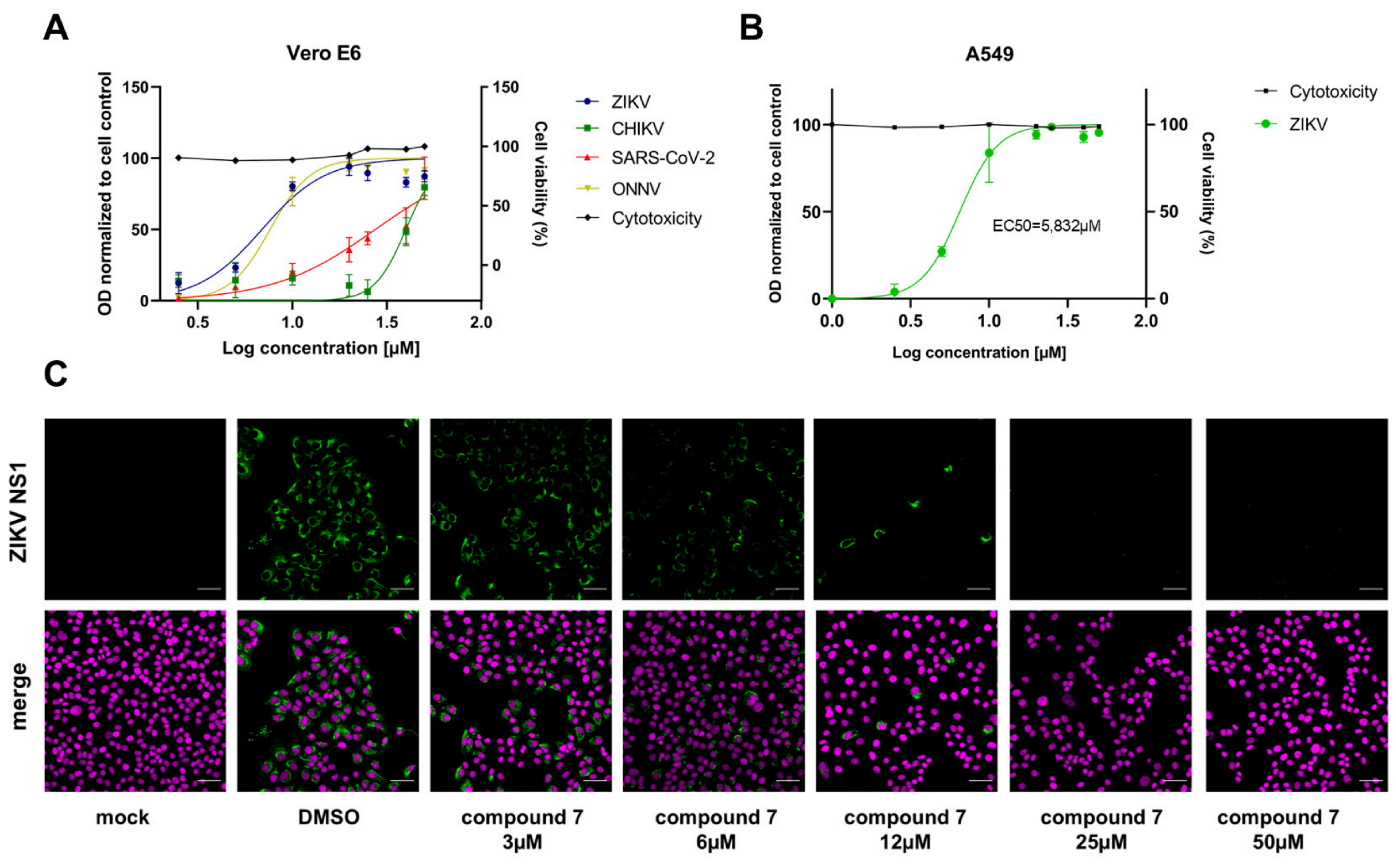
GPA derivatives exhibit a broad range of virucidal effects. (**A**) Compound **7** inhibits all four viruses in Vero E6 cell line. Cell viability was measured and a non-linear regression curve was fitted to our data. (**B**) The inhibitory effect of compound **7** against ZIKV (MOI: 1) on the A549 cell line, where EC_50_ was calculated by non-linear regression analysis. (**C**) Antiviral activity of compound **7** was determined using an immunofluorescence assay. Cells were infected with ZIKV with MOI: 5, fixed 24 hpi. The nuclei were stained with DAPI (magenta), and Zika infection was visualized with a ZIKV NS1 antibody (green). The images were taken with Zeiss LSM 710 Confocal Microscope. Data represent the mean ± SEM of at least three independent experiments. Scale bar: 50 µm.

In our study, we examined the antiviral activity and cytotoxicity of the native antibiotics teicoplanin, vancomycin, ristocetin and their deglycosylated aglycones and pseudoaglycones and semisynthetic hydrophobic derivatives (compound **1–8**) (overall 14 glycopeptide antibiotic derivatives; see the structures in the Supplementary Materials). The studied compounds showed a broad range of inhibitory effects. Vero E6 cells were infected with ZIKV (MOI: 1), SARS-CoV-2 (MOI: 0.1), CHIKV (MOI: 0.1), and ONNV (MOI: 0.1). Due to differences in viral replication dynamics, a higher MOI was used for ZIKV. Among the natural glycopeptide antibiotics and their aglycone/pseudoaglycone derivatives, only the native teicoplanin complex showed some antiviral activity. Most of the semisynthetic hydrophobic teicoplanin derivatives (compounds **1–7**) did not exhibit any cytotoxic effects at concentrations below 50 µM, while demonstrating noticeable antiviral activity. The hydrophobic ristocetin derivative **8** showed an inhibitory effect against all tested viruses but was also slightly cytotoxic. It is important to note that we only tested the virus-inhibitory effects of the compounds at concentrations where no cytotoxic effects were observed. During the experiments, we concurrently examined the GPAs on both infected and uninfected cells where, in two cases, inhibitory effects against all four viruses were observed (Table 1). The compounds were tested against different viruses in a seven-element concentration series. Microscopic observation was used to monitor cytopathic effects (CPEs), which refer to structural changes in host cells caused by viral infection, and to assess how these changes are influenced by antiviral treatment. Cell viability was assessed using the MTT assay, and EC_50_ (half-maximal effective concentration) and CC_50_ (50% cytotoxic concentration) values were calculated from optical density (OD) measurements. Selectivity index (SI) values were also determined, and only those above 10 were considered suitable for further investigation. In the case of ZIKV, the assay was also performed on the A549 cell line with compound **7**. The EC_50_ value (5.832 µM) was very similar to the result obtained on Vero E6 (5.18 µM) (Figure 2). No cytopathic effects were observed for compounds **2**, **3**, **4**, **6** and **7**, after five days of ZIKV infection, at the highest concentrations, where compounds caused no cytotoxicity (Supplementary Figure S4).

### 3.3. Compound ***7*** Reduces the Viral RNA Level During the Early Stages of ZIKV Infection

**Figure 3:**
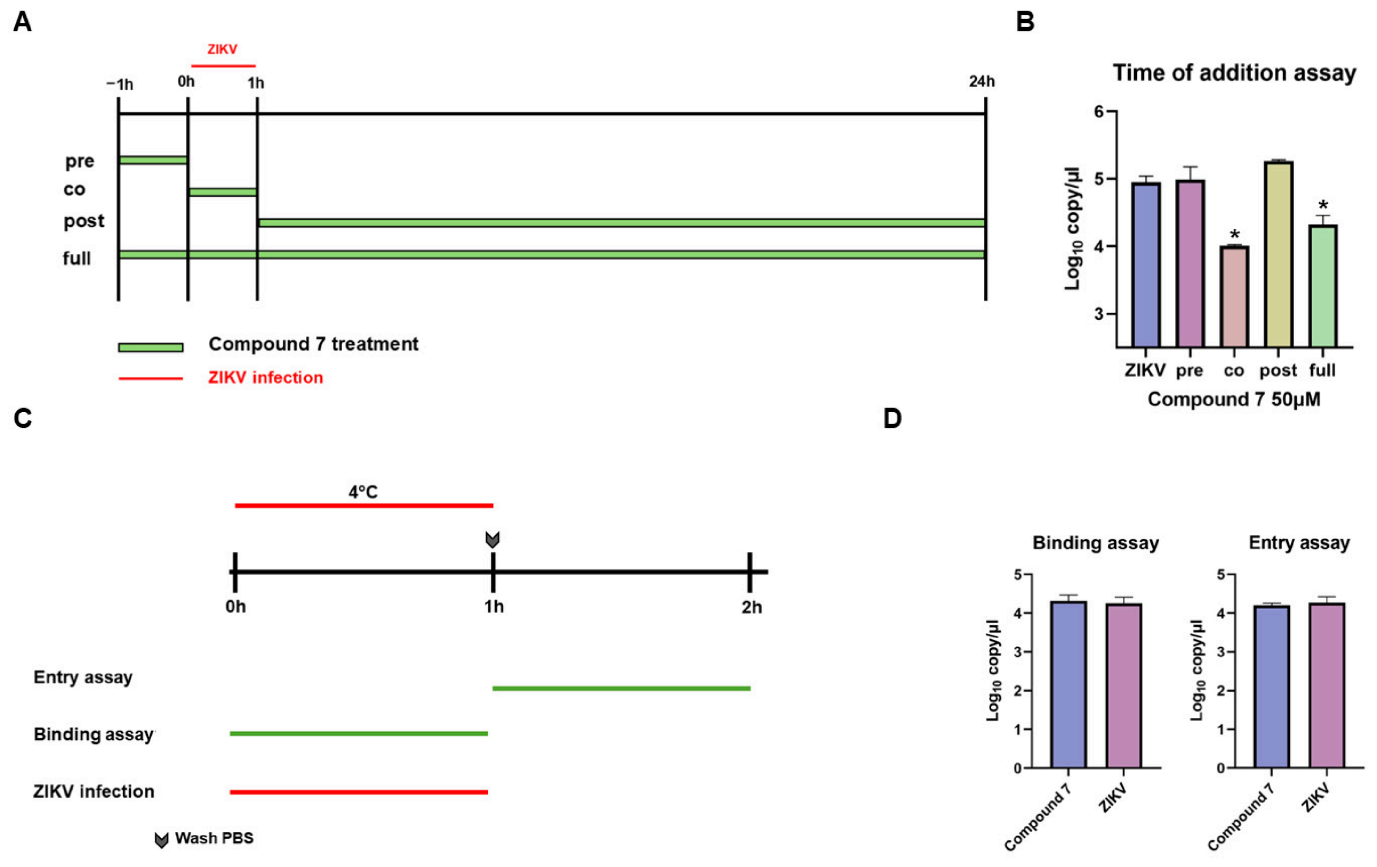
Early-stage antiviral activity of compound 7 against ZIKV. (**A**) Schematic representation of the time-of-addition assay. Vero E6 cells were infected for 1 h at MOI of 1. Compound **7** (50μM) was added at different time points. Twenty-four hours post-infection, treatment was terminated and viral RNA levels were measured. (**B**) Result of the time-of-addition assay. The viral RNA expression was measured by qRT-PCR. Statistical significance was determined using Student’s *t*-test (* *p* < 0.01). Bar graphs represent the mean. (**C**) Results of binding and entry assay showed no significant differences. (**D**) Schematic representation of the binding and entry assays. Vero E6 cells were infected for 1 h at 4 °C at MOI of 1. Each experiment was repeated independently at least three times (*n* = 3, ±SEM).

After establishing the potent anti-ZIKV activity of compound **7** we performed a time-of-addition assay to identify at which stage of the viral replication cycle the compound possesses inhibitory activity. We divided the samples into five groups based on the timing of treatment: no-treatment (ZIKV), pre-treatment for 1 h (pre), co-treatment (co), post-treatment for 1 h (post), and full treatment (full) (Figure 3A). Twenty-four hours post-infection (pi), we isolated viral RNA from the supernatant and quantified it using qRT-PCR. The virus was added at MOI of 1 and for the treatment 50 µM compound **7** was used. The results indicated significant differences in viral RNA levels compared to the no-treatment group only in the case of co-treatment, leading us to conclude that the compound probably exerts its effects during the early stages of infection (Figure 3B).

### 3.4. Compound ***7*** Inactivates ZIKV Particles Without Detectable Virion Destabilization

To gain a more precise understanding of the mechanism of action of compound **7**, binding and entry assays were performed. Previous time-of-addition experiments indicated that compound **7** exerts its antiviral effect at an early stage of infection. Therefore, we investigated whether the compound interferes with viral attachment or entry. In the binding assay, infection and treatment were conducted simultaneously for 1 h at 4 °C. This temperature prevents viral internalization and therefore allows selective evaluation of virus attachment to the cell surface [29]. For the entry assay, cells were first infected for 1 h at 4 °C to allow viral attachment, washed twice with PBS to remove unbound virus, and subsequently treated with compound **7** during incubation at 37 °C for 1 h to permit viral entry (Figure 3D). Vero E6 cells were infected at a multiplicity of infection (MOI) of 1. After incubation, cells were washed with PBS prior to RNA extraction, and viral RNA levels were quantified by qRT-PCR. No significant differences were observed between treated and control groups in either assay (Figure 3D).

**Figure 4:**
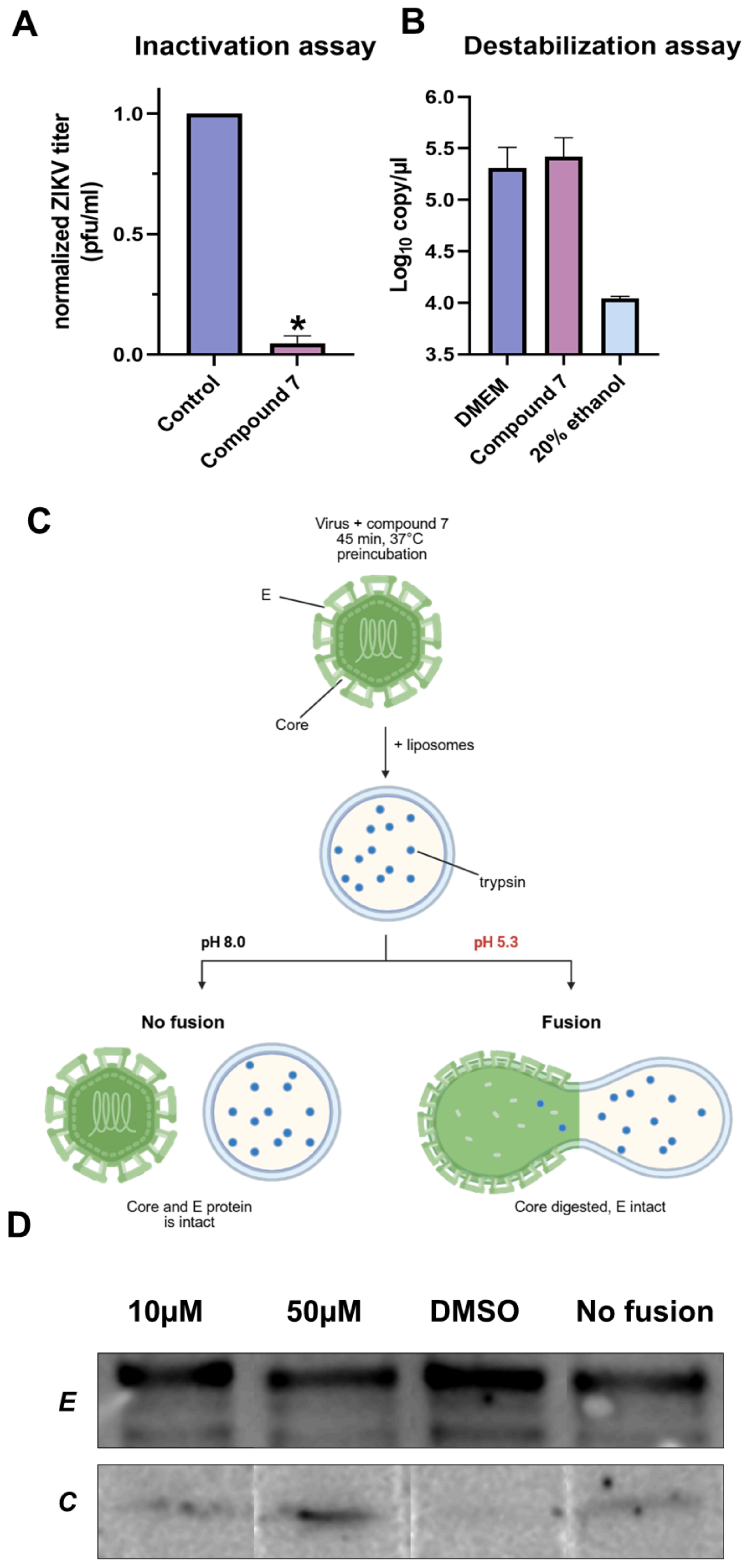
Compound **7** acts in a cell-free environment. (**A**): Virion inactivation assay. Plaque assays were performed in three independent experiments. The results of plaque assays were normalized to the virus stock titer. The treated stock has significant differences compared to the non-treated stock. The significance was calculated by Student’s *t*-test * *p* < 0.01. (**B**): Destabilization assay. The treated virus stock has no significant differences compared to DMEM-treated stock. These results indicate that compound **7** does not cause detectable disruption of virion integrity. Each experiment was repeated independently at least three times (n = 3, ±SEM). (**C**): Liposome-based capsid protection assay used to evaluate ZIKV membrane fusion. In this system, synthetic liposomes encapsulating trypsin are used to detect fusion events. Upon low-pH-induced fusion between the viral envelope and the liposome membrane, the viral capsid becomes exposed to the liposomal lumen, allowing trypsin to access and digest the capsid (*C*) protein, while the envelope (*E*) protein remains intact and serves as a control. The “No fusion” control represents samples maintained under neutral pH conditions, without low-pH treatment required to trigger membrane fusion. (**D**): Western blot analysis of ZIKV envelope (*E*) and capsid (*C*) proteins following treatment with compound **7**. Protection of the capsid protein from trypsin digestion indicates that compound **7** inhibits low-pH-triggered virus–liposome fusion. Data shown are representative of at least two independent experiments.

These results indicate that compound **7** does not interfere with viral attachment or entry. Given that the compound acts at an early stage of infection, this raised the possibility that it may directly inactivate the virion. To test this hypothesis, a virion inactivation assay was performed. The virus stock was pre-incubated with 50 µM of compound **7** at 37 °C for 2 h, followed by a 100-fold dilution of the virus–compound mixture to eliminate residual compound from the system. Infectious virus titers were then determined by plaque assay. Treatment with compound **7** reduced viral titers by approximately one order of magnitude compared with the untreated control (Figure 4A), indicating direct inactivation of virions in a cell-free environment. To determine whether this effect resulted from structural disruption of the virion, a destabilization assay was conducted. Virus stock (approximately 5 × 10^6^ PFU/mL) was incubated with 50 µM of compound **7** for 1 h at 37 °C, followed by treatment with RNase A for 1 h at 37 °C. Proteinase K was subsequently added to degrade RNase A prior to RNA extraction. Viral RNA levels were quantified by qRT-PCR. As a positive control, 20% ethanol was used, whereas DMEM served as a negative control. No significant reduction in viral RNA copy number was observed compared with the negative control (Figure 4B). These findings indicate that compound **7** does not cause detectable disruption of virion integrity. To further investigate the mechanism underlying this effect, we performed a liposome-based capsid protection assay to assess the impact of compound **7** on low-pH-triggered membrane fusion. In this assay, fusion between the viral envelope and trypsin-containing liposomes exposes the viral capsid protein to proteolytic digestion, while the envelope (E) protein remains intact and serves as an internal control. Western blot analysis of ZIKV envelope (E) and capsid (C) proteins revealed that exposure of the virus–liposome mixture to acidic pH resulted in digestion of the capsid protein in the fusion control. In contrast, treatment with compound **7** preserved the capsid signal, indicating protection from trypsin digestion (Figure 4C,D). These results indicate that compound **7** inhibits low-pH-induced virus–liposome fusion Together with the inactivation assay results, these findings suggest that compound 7 directly targets ZIKV virions and interferes with the membrane fusion step required for ZIKV entry. Whether similar mechanisms contribute to the antiviral activity against CHIKV, ONNV, or SARS-CoV-2 remains unknown.

### 3.5. Compound ***7*** Induces Mild Phospholipidosis in a Concentration-Dependent Manner

As drug-induced phospholipidosis has been proposed as a potential explanation for the apparent antiviral activity of some compounds in vitro [51], we examined whether compound **7** induces phospholipid accumulation in treated cells. Compound **7** induced a weak phospholipidosis response that was detectable from 2.5 µM and increased in a concentration-dependent manner. However, the magnitude of the response remained substantially lower than that observed with the positive control propranolol (30 µM) (Supplementary Figure S5).

### 3.6. Mechanistic Investigation of the Effect of Compound ***7*** In Silico

**Figure 5:**
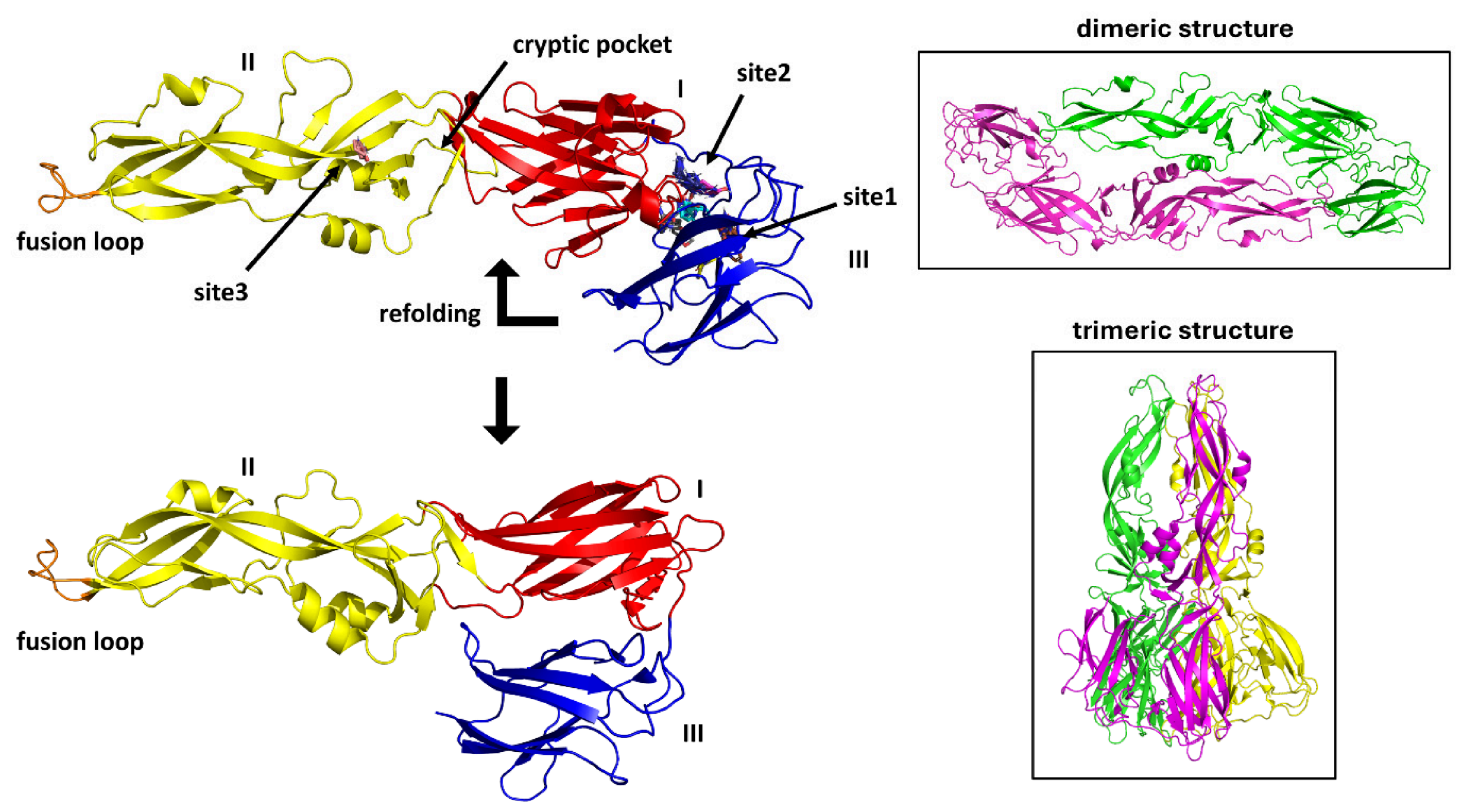
Reorganization of the unbound, dimeric form into a membrane-bound, trimeric form is a key step in the membrane fusion mechanism of flaviviruses, illustrated with the respective structures of the Zika (5LBV) and Dengue (1OK8) E proteins, with the full dimer and trimer structures shown in the insets. The binding sites detected by FTMap are shown on the surface of the Zika structure. (See Supplementary Figure S1 for more detail.)

The ZIKV envelope protein (E protein) contains three domains [52]: a central β-barrel domain (domain I), a long finger-like domain (domain II) and an immunoglobulin-like domain (domain III) at the C-terminus (Figure 5), and there is an additional stem region and transmembrane anchor region of the protein at the C-terminus, which is not shown. Infected cells internalize ZIKV particles by envelope-protein-mediated endocytosis [53]. Before uptake, the envelope protein of the virus sits on the viral surface in the form of antiparallel dimers, with their fusion loop buried. The dimers dissociate with the lower pH of the endosome, which exposes the fusion loop region of the monomers. The fusion loop can integrate into the endosomal membrane, which causes the formation of E protein trimers [54]. Then, by proposedly irreversible conformational changes, the trimers fuse the endosomal and viral membranes, releasing the genetic content of the virus inside the cytosol. This is illustrated by the E protein trimer structure of the DENV, whose domain III closes itself onto domain I, with its C-terminus pushed towards the fusion loop (Figure 5). Also, there is a slight rotation of domain II towards domain I.

To investigate the mechanistic details of the effect of compound **7** upon the function of the envelope protein, we utilized state-of-the-art binding site detection and ligand docking tools, as well as the X-ray crystal structures of the Zika virus E protein dimer from the Protein Data Bank (PDB IDs 5JHM [52] and 5LBV [54]).

First we mapped possible binding sites on the surface of every deposited chain in the two complexes using the FTMap protocol [31,32]. FTMap is based on the conformational sampling of small, organic probe molecules on the protein’s surface, in order to identify energetically favorable regions, so-called hotspots, that are particularly suitable for small-molecule or macromolecular ligand binding. Here, FTMap identified three binding sites (Figure 5): Site1 is found at the interface between domains I and III, Site2 is found only on the chains of the 5LBV structure in the region connecting domains I and III near the glycan loop (which is not present in structure 5JHM), while Site3 appears as a weaker, shallow binding pocket on domain II, near its interface with domain I, near a possible cryptic, hydrophobic pocket, hidden by a β-hairpin loop (residues 274–286 in 5JHM and 5LBV) [55].

While we did not find any prior studies describing Site1, it apparently disappears during the dimer–trimer reorganization upon membrane fusion (Supplementary Figure S2). To corroborate this, we mapped the surface of the trimeric DENV E protein structure (PDB ID:1OK8) with FTMap where, indeed, this site was not found (Supplementary Figure S2). Therefore, we propose that ligand binding at this location could stabilize the dimeric form and thereby inhibit dimer–trimer conversion and, consequently, membrane fusion.

Site2 was identified by Sharma and co-workers by computational means and described as the likely binding site of a natural compound and as a further inhibitor discovered by virtual screening, found to inhibit ZIKV infection in vitro [56,57,58]. The proposed mechanism of action is that ligand binding reduces the flexibility of the linker that connects domains I and III (residues 298–305), which in turn is needed for domain rearrangement and membrane fusion [59].

The cryptic pocket next to Site3 was detected experimentally on the dengue virus E protein by co-crystallizing it with the detergent *n*-octyl-β-D-glucoside (β-OG) [55]. Several dengue membrane fusion inhibitors were found to bind to this site and it was proposed as a general binding site for antiflaviviral drugs, due to several conserved residues found here [60]. Here, we hypothesized that the poly-fluorinated tail region of compound **7** could bind to this cryptic site similarly to β-OG, while the multicyclic core could fit the shallower binding site identified by FTMap.

To assess the binding mode of compound **7**, we used Schrödinger Glide (and for Site3, Induced Fit Docking, see Section 2) to dock the molecule to the three binding sites found. We ranked the resulted docking poses for each chain based on their Glide scores and selected the best 20 ligand–protein complexes, which were finally evaluated by MM/GBSA dG_bind_ scores and visual inspection (Supplementary Table S1). We should stress that the MM/GBSA dGbind scores only serve the purpose of comparing alternative binding modes and are not to be considered as reliable estimators of the true free energies of binding.

**Figure 6:**
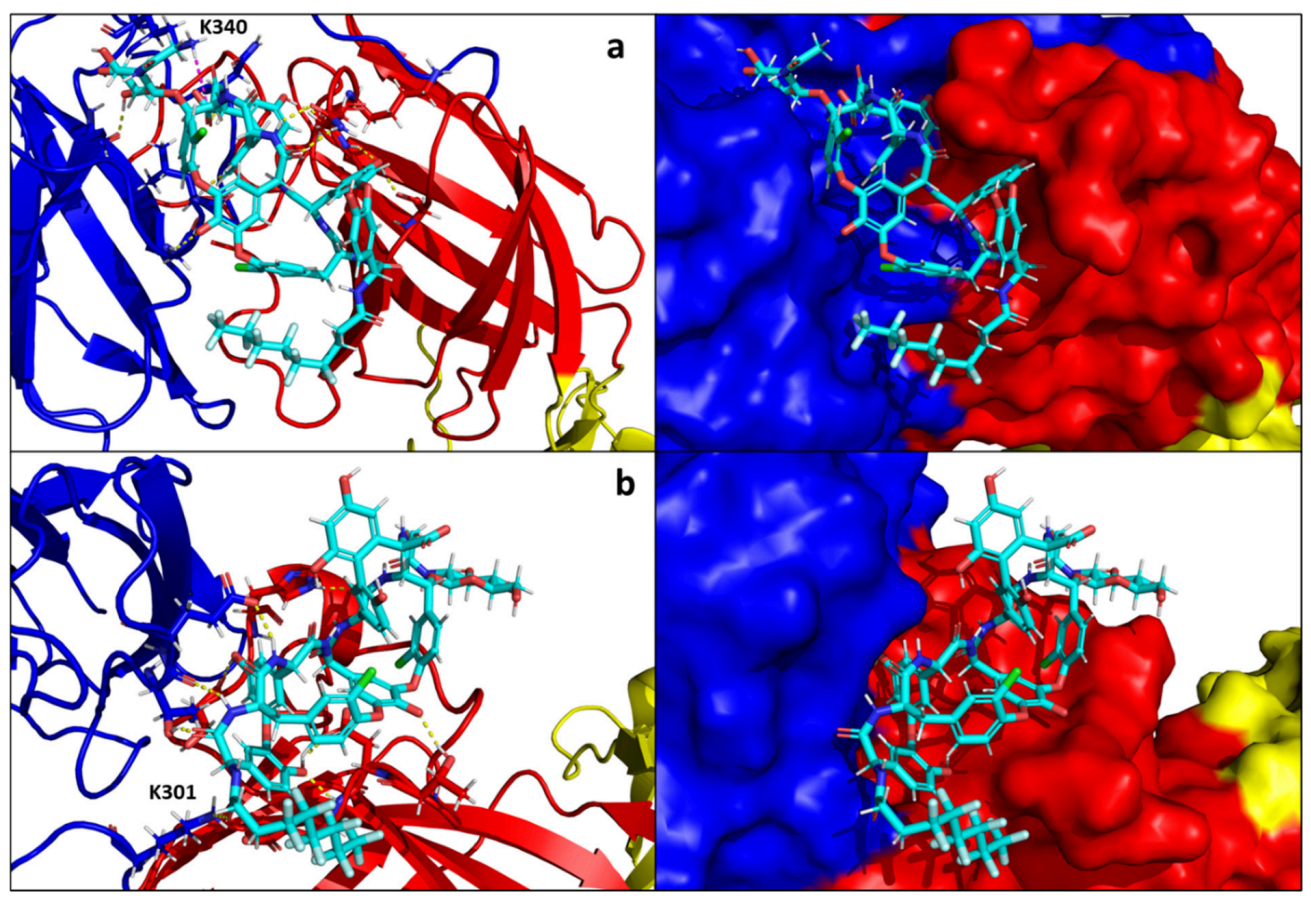
Modeled binding poses of compound **7** in the different binding sites ((**a**): Site1, (**b**): Site2) with cartoon (**left**) and surface (**right**) representations, colored according to the different domains (domain I: red, domain II: yellow, domain III: blue). Non-covalent interactions are shown with dotted lines (yellow for hydrogen bonds, purple for salt bridges).

The different binding modes predicted for compound **7** are shown in Figure 6. We were not able to produce a viable binding pose for Site3, so we excluded this binding site from further analysis. Of the remaining two binding pockets, Site1 produced a better MM/GBSA score (Supplementary Table S1) and showed a tighter complementarity with the pocket. Here, several contacts are detected with residues from domains I and III, including a salt bridge with the side chain of K340. At Site2, the calculated dG_bind_ score is almost half of that at Site1 and, focusing on the linker loop between domains I and III (needed for trimer formation), the modeled pose most notably shows a hydrogen bond interaction with K301. However, due to the highly flexible nature of its H-bonding partner (amide linker connecting the fluorinated tail of compound **7** to its core unit), we cannot safely assume that this binding mode would be strong enough to exert any functional effect by interacting with this loop. This was further corroborated by equilibrium MD simulations of the predicted binding modes of **7**, showing overall stability for the binding mode in Site1, and multiple dissociation events in Site2 (Supplementary Figure S3). Therefore, we propose Site1 as the main binding site for compound **7**, with the inhibition of the E protein dimer–trimer conversion as the basis for its detected in vitro antiviral activity.

## 4. Discussion

Zika virus continues to infect tens of thousands of people worldwide each year. Despite decades of familiarity with the virus, vaccine developments against it are currently only in Phase 1 or 2 clinical trials [8]. Numerous compounds have proven effective in vitro against the virus, but currently, no drugs are available for treating the infection. These limitations highlight the importance of identifying new potential drug candidates and understanding their mechanism of action. Compounds exhibiting broad antiviral activity are particularly valuable for therapeutic development. Therefore, in addition to ZIKV, the antiviral potential of the tested compounds was evaluated against several unrelated viruses, including chikungunya virus (CHIKV), o’nyong-nyong virus (ONNV), and SARS-CoV-2. Alphaviruses were included in the study because compounds of this class have not previously been investigated against these viruses. GPA compounds and their derivatives have previously been reported to exhibit antiviral activity against several viruses, including DENV, EBOV, HCV, etc. [17,21,23]. For many compounds, they have been shown to inhibit entry, but the mechanism of action may differ depending on the virus and the type of side chain of the compound [18,20,23]. For example, our earlier studies have identified host Cathepsin L and (to a smaller extent) the viral main protease as the main targets of related GPAs for SARS-CoV-2 inhibition [16,25].

The MTT cell viability assay was used to determine the EC_50_ values of eight semisynthetic derivatives, along with the natural glycopeptides teicoplanin, ristocetin, and vancomycin, and their corresponding aglycone/pseudoaglycone derivatives, against ZIKV, CHIKV, ONNV, and SARS-CoV-2. Seven of these semisynthetic compounds were shown to be effective against ZIKV, demonstrating the general viability of this chemical platform against emerging viral threats.

In our 2020 study [24], we evaluated only a limited number of teicoplanin and vancomycin derivatives against a diverse panel of viruses, and the vancomycin-derived structures tested were mostly inactive. In the present work, we not only expanded the range of viruses tested but also made efforts to determine the antiviral mechanism of action of the derivative most effective against ZIKV. Furthermore, we significantly expanded the structural diversity to establish detailed structure–activity relationships. To achieve this, we included a third antibiotic backbone, ristocetin, in the study, while also systematically modifying both the nature of the linker and the length of the perfluoroalkyl side chains. The complete inactivity of compound **5** showed that the long amphiphilic tetraethylene glycol linker was detrimental to the antiviral effect. In line with our previous results [59], we found that, although the peptide backbones of the glycopeptide antibiotics are very similar, there is a significant difference in the antiviral activity and selectivity of their derivatives, which is clearly shown by the different activity profiles of the identically substituted pair of compounds, teicoplanin derivative **6** and ristocetin derivative **8**.

We and others have amply demonstrated that a lipophilic/hydrophobic group must be attached to the glycopeptide backbone to confer antiviral activity to the parent compounds [16,20,22,23,26]. This is confirmed by the present study, as glycopeptide aglycone/pseudoaglycone derivatives did not exhibit any antiviral activity on their own. Our previous studies showed that the introduction of 6–18 carbon alkyl/aryl groups connected to the glycopeptide backbone through linker moieties of varying lengths resulted in the best antiviral effects, but this effect was often accompanied by cytotoxicity, which was attributed to the membrane-disrupting properties of the lipophilic groups [26,61]. The introduction of perfluoro groups, which are both lipo- and hydrophobic, instead of the lipophilic alkyl/aryl groups, eliminated the cytotoxicity of the compounds, while in most cases the antiviral properties were preserved [24,25]. Although the role of the perfluoro groups is not precisely elucidated, they probably facilitate cell entry either by penetrating the membrane without perturbing it or by reducing the overall hydrophilicity of the compound.

The antiviral activities were influenced by both the size of the perfluoro group and the linker moiety. The predicted binding mode did not clearly identify any direct interactions between the target and the perfluorinated tail, which supports our notion that the effect on antiviral activity is mainly driven by the change in permeability, as noted above. Against ZIKV, the newly introduced perfluorohexyl-containing derivatives **4** and **7**, which contain a short linker, showed the highest antiviral activity, with an EC_50_ value of 5 μM; in these derivatives, the total length of the hydrophobic chain is nine and 13 atoms, respectively. The perfluorooctyl compound **6**, in which the total chain length of the modifying group is 11 atoms, showed almost the same good effect. Importantly, our results with these derivatives demonstrate that neither the triazole ring nor the ethylene glycol spacer is absolutely necessary for the antiviral effect. Slightly lower antiviral activities of ~11 μM were shown by compounds **1–3**, which contained hydrophobic chains of 18, 16, and 14 atoms, while compound **5**, which contains a 26-atom hydrophobic group, was completely inactive. The optimal structure against ZIKV is therefore a short, 9–13-atom hydrophobic modification with a perfluorohexyl/octyl group, with a small increase in the size of the modifying group resulting in a decrease in activity and a significant increase in size resulting in a loss of activity.

Several of the synthesized compounds have broad-spectrum antiviral effects such as compound **7** which proved effective against ONNV, CHIKV, and SARS-CoV-2 at low micromolar concentrations. We further investigated the antiviral activity of compound **7** to better understand its mechanism of action against ZIKV. First, a time-of-addition assay was performed to determine the stage of the viral life cycle affected by the compound. The results indicated that compound **7** acts during the early stages of infection, as inhibitory activity was only observed when the compound was present during the initial phase of the infection process. No significant inhibition was detected when the compound was applied either before infection or after viral entry. Further confirmation came from the binding and entry assay demonstrating that compound **7** does not inhibit viral binding or entry. We next examined whether compound **7** could act directly on the virion. In the virion inactivation assay, pre-incubation of the virus with compound **7** resulted in a marked reduction in viral infectivity in a cell-free environment. However, the destabilization assay showed no increase in viral genome accessibility, indicating that the observed loss of infectivity was not caused by disruption of virion integrity. Then we performed a liposome-based capsid protection assay, which provided important additional support for our hypothesis. In this system, low-pH-induced fusion between the viral envelope and trypsin-containing liposomes exposes the viral capsid protein to proteolytic digestion, while the envelope protein remains intact. Western blot analysis showed that, in the presence of compound **7**, the capsid protein was protected from digestion under fusion-permissive conditions. These findings indicate that compound **7** inhibits low-pH-triggered virus–liposome fusion and strongly support the hypothesis that the compound acts by interfering with the fusion process rather than by disrupting virion integrity. Whether similar mechanisms contribute to the antiviral activity observed against CHIKV, ONNV, and SARS-CoV-2 remains to be determined. Most mechanistic experiments were performed at a single concentration (50 μM), selected as the highest non-cytotoxic concentration to maximize detection of antiviral effects. Evaluation at additional concentrations would further strengthen the mechanistic characterization. Drug-induced phospholipidosis has been reported to contribute to the apparent antiviral activity of a number of compounds in cell culture systems. This phenomenon has attracted significant attention in antiviral drug research, as the accumulation of phospholipids in endolysosomal compartments can disrupt the intracellular transport pathways necessary for viral entry and replication, which may lead to false-positive antiviral results under in vitro conditions [51]. Therefore, the phospholipidosis-inducing potential of compound **7** was also evaluated. Although compound **7** induced phospholipid accumulation in a concentration-dependent manner, the observed response was markedly weaker than that produced by the positive control propranolol. Moreover, only limited phospholipidosis was detected at concentrations associated with antiviral activity. In addition, compound 7 partially reduced ZIKV infectivity in a cell-free inactivation assay and inhibited low-pH-triggered ZIKV–liposome fusion, indicating that direct antiviral activity contributes to its anti-ZIKV effect independently of cellular alterations. However, the incomplete reduction in infectivity observed in the cell-free assay suggests that direct virion targeting alone is unlikely to fully account for the antiviral activity of compound **7**, and additional mechanisms cannot currently be excluded. Collectively, these findings support a direct antiviral mode of action against ZIKV, while a contribution of phospholipidosis-related cellular effects and other mechanisms cannot be excluded.

Using bioinformatic analysis, we further explored the possible molecular basis of this effect. FTMap scanning of the viral E protein identified several regions with potential affinity for compound **7**. We identified three potential binding sites, all located near structural regions involved in the conformational rearrangements required for membrane fusion between the viral envelope and the endosomal membrane. Although the docking results do not provide direct proof of binding, all experimental observations preceding this analysis are consistent with this model. Nevertheless, the proposed binding mode remains hypothetical and requires further experimental validation.

Compound **7** was also tested in the human A549 cell line, where it showed an EC_50_ value similar to that observed in Vero E6 cells. This indicates that the antiviral effect is not restricted to a single cell type. Overall, our results suggest that compound **7** acts through a mechanism that differs from those previously described for related glycopeptide antibiotic derivatives. In the case of ZIKV, the available data support a model in which the compound acts directly on the virion and interferes with the membrane fusion step required for infection, most likely through interaction with the viral E protein in a manner that is independent of the cellular environment. To our knowledge, this type of fusion-inhibitory mechanism has not previously been described for this compound family. Despite the ability of compound **7** to act directly on the virus in a cell-free environment, other GPA derivatives with similar lipophilic side chains have been found to act inside cells. From these we conclude that, despite the large size of our molecule, it may be able to enter the cell [18,62].

The compounds we examined warrant further investigation, contributing to the development of new antiviral drugs. These compounds not only have inhibitory effects against ZIKV but also various alphaviruses and certain coronaviruses. The development of broad-spectrum antiviral drugs is essential for the treatment of current and future expected epidemics.

## 5. Conclusions

In our study, we have identified glycopeptide antibiotic derivatives with potent antiviral activity against Zika virus as well as several other epidemiologically important RNA viruses. Mechanistic studies performed with ZIKV suggest that compound 7 interferes with the membrane fusion step of infection, most likely through direct interaction with the virion, independent of host cellular factors. These findings substantially extend our previous work by expanding both the structural diversity of the investigated glycopeptide derivatives and the mechanistic understanding of their antiviral activity, while further supporting the potential of structurally optimized natural product derivatives as promising broad-spectrum antiviral agents for future investigation.

## Figures and Tables

**Table 1:** Inhibitory effect of GPA derivatives against various viruses based on the CPE inhibition assay on Vero E6 cell line.

			ZIKV		SARS-CoV-2		CHIKV		ONNV	
Compounds	MCC (µM)	CC_50_ (µM)	EC_50_ (µM)	SI	EC_50_ (µM)	SI	EC_50_ (µM)	SI	EC_50_ (µM)	SI
Teicoplanin	>60	279.90	n.a.	-	33.88	8.26	86.23	3.25	n.a.	-
**1**	>50	148.80	24.27	6.13	20.01	7.44	n.a.	-	n.a.	-
**2**	>40	195.30	11.77	16.59	17.48	11.17	n.a.	-	n.a.	-
**3**	>50	198.20	11.23	18.56	53.21	3.72	35.92	5.52	n.a.	-
**4**	>50	249.80	5.26	47.49	10.50	23.79	28.07	8.90	n.a.	-
**6**	>60	174.60	6.14	28.44	4.70	37.15	61.07	2.86	n.a.	-
**7**	>60	203.30	5.18	39.25	10.29	19.76	6.49	31.33	5.37	37.86
**8**	>20	62.63	42.3	1.48	9.88	6.34	14.04	4.46	39.50	1.59

Abbreviations: MCC—minimal cytotoxicity concentration, compound concentration producing minimal changes in cell morphology, as estimated by microscopy; EC_50_—half maximal effective concentration; SI—selectivity index; CC_50_—50% cytotoxic concentration; n.a.—not active.

## Data Availability

The original contributions presented in this study are included in the article and/or Supplementary Material. Further inquiries can be directed to the corresponding authors.

## Supplementary Materials

The following supporting information can be downloaded at: https://www.mdpi.com/article/10.3390/pharmaceutics18070879/s1. This file contains detailed molecular modelling protocols; light microscopy images of the infected cells; phospholipidosis assay results; chemical structures of the studied glycopeptide antibiotic derivatives; and NMR analysis of compounds **2**, **7**, and **8**, as well as the NMR spectra of the synthetic intermediates.

## Abbreviations

A549	human lung carcinoma cell line
BAIB	bis(acetoxy)iodobenzene
CC_50_	50% cytotoxic concentration
CHIKV	chikungunya virus
CPE	cytopathic effect
DENV	dengue virus
DMEM	Dulbecco’s modified Eagle medium
EC_50_	half-maximal effective concentration
E protein	envelope protein
FTMap	fragment-based mapping
GBS	Guillain–Barré syndrome
GPA	glycopeptide antibiotic
HCV	hepatitis C virus
HIV	human immunodeficiency virus
HSV	herpes simplex virus
IAV	influenza A virus
IBV	influenza B virus
MALDI-TOF MS	matrix-assisted laser desorption/ionization time-of-flight mass spectrometry
MCC	minimal cytotoxic concentration
MERS-CoV	Middle East respiratory syndrome coronavirus
MM/GBSA	molecular mechanics/generalized Born surface area
MOI	multiplicity of infection
NS1	non-structural protein 1
NHS	N-hydroxysuccinimide
OD	optical density
ONNV	o’nyong-nyong virus
PBS	phosphate-buffered saline
PFU	plaque-forming unit
qRT-PCR	quantitative reverse transcription polymerase chain reaction
RNA	ribonucleic acid
RSV	respiratory syncytial virus
SARS-CoV	severe acute respiratory syndrome coronavirus
SARS-CoV-2	severe acute respiratory syndrome coronavirus 2
SEM	standard error of the mean
SI	selectivity index
TBEV	tick-borne encephalitis virus
TEMPO	tetramethylpiperidine 1-oxyl
THF	tetrahydrofuran
WNV	West Nile virus
WHO	World Health Organization
YFV	yellow fever virus
ZIKV	Zika virus
