# Supplementary material for: Fusion Inhibition of Zika Virus Entry by a Teicoplanin Pseudoaglycone Derivative with Broad Antiviral Activity
Source: Pharmaceutics. 2026 Jul 17;18(7):879. doi: 10.3390/pharmaceutics18070879 (PMC13415800; doi:10.3390/pharmaceutics18070879)
Supplement: Supplementary file 1 [file pharmaceutics-18-00879-s001.zip › pharmaceutics-4388386-supplementary.pdf]

pharmaceutics

*Supporting Information*

# Semisynthetic glycopeptide antibiotics with antiviral activity against Zika virus and other emerging viruses

**Zoltán Kopasz [1,2] ‡, Ilona Bereczki [1,3] ‡, Krisztina Leiner [1,2], Henrietta Papp [1] †, Eszter Boglárka Lőrincz [3,6], Levente Sipos-Szabó [4,5], Kornélia Bodó [1], Eszter Szabó [1], Mónika Madai [1], Brigitta Zana [1], Réka Erdei [7,8], Gyula Batta [7], Tamás Kovács-Öller [2,10], , Zoltán Varga [11,12], Dávid Bajusz [5], Gábor Kemenesi [1,2], Anikó Borbás [1,3,9]* and Anett Kuczmog [1,2]***

[1] National Laboratory of Virology, Szentágothai Research Centre, University of Pécs, Pecs, Ifjúság útja 20, 7624, Hungary
[2] Faculty of Sciences, Institute of Biology, University of Pécs, Pecs, Ifjúság útja 6, 7624, Hungary
[3] Department of Pharmaceutical Chemistry, University of Debrecen, Debrecen, Egyetem tér 1, 4032, Hungary
[4] Department of Organic Chemistry and Technology, Budapest University of Technology and Economics, Budapest 1111, Hungary
[5] Medicinal Chemistry Research Group and Drug Innovation Centre, HUN-REN Research Centre for Natural Sciences, Magyar tudósok krt. 2, 1117 Budapest, Hungary
[6] Doctoral School of Pharmaceutical Sciences, University of Debrecen, Egyetem tér 1, H-4032 Debrecen, Hungary
[7] Department of Organic Chemistry, University of Debrecen, H-4032 Debrecen, Hungary;
[8] Doctoral School of Chemistry, University of Debrecen, Egyetem tér 1, H-4032 Debrecen, Hungary
[9] HUN-REN–UD Molecular Recognition and Interaction Research Group, University of Debrecen, Debrecen, H-4032, Hungary
[10] János Szentágothai Research Centre, University of Pécs, Pecs, Ifjúság útja 20, 7624, Hungary
[11] Biological Nanochemistry Research Group, Institute of Materials and Environmental Chemistry, HUN-REN Research Centre for Natural Sciences, Magyar tudósok körútja 2, H-1117 Budapest, Hungary
[12] Department of Physical Chemistry and Materials Science, Faculty of Chemical Technology and Biotechnology, Budapest University of Technology and Economics, Műegyetem rkp. 3., H-1111 Budapest, Hungary
*kuczmog.anett@pte.hu
*borbas.aniko@pharm.unideb.hu

## Table of contents:

1. Detailed molecular modeling protocols S-03
2. Light microscopy images from the infected cells S-09
3. Phospholipidosis assay result S-09
4. Structures of the studied glycopeptide antibiotic derivatives S-10
5. NMR analysis of compounds 2, 7 and 8 S-14
6. NMR spectra of the synthetic intermediates S-26

## Supplementary Note 1. Detailed molecular modeling protocols

*Protein structure preparation*

The structures were refined with the Protein Preparation Wizard of the Schrödinger suite with default settings (Sastry et al., 2013). Here, missing heavy atoms and hydrogen atoms were added, missing residues and loops were modeled, and the structure was relaxed in the OPLS4 force field (Lu et al., 2021). All non-protein entries and waters were removed from the structures. There was a longer loop missing from the deposited 5JHM structure on both chains (147-161), which was further refined using the Prime loop refinement application (Jacobson et al., 2004).

 https://doi.org/10.3390/pharmaceutics18070879

*Ligand preparation*

For the docking studies, we generated the model of compound **7** with Schrödinger Ligprep, and here the whole 3D atomic model of the molecule was built with the most probable protomer assigned with Epik at physiological pH (Johnston et al., 2023). Next we ran a conformational sampling search with the Macromodel mixed torsional and low-mode sampling protocol on default settings in the OPLS4 force field (Mohamadi et al., 1990; Sastry et al., 2013). Here, the molecule's conformational space is explored by changing the conformation by varying Monte Carlo or low-mode steps. The lowest energy conformations are retained and used for further conformer generation until convergence. Conformers with less than 0.5 root mean squared deviation (RMSD) between identical atoms in the macrocyclic core after alignment were filtered out.

Every docking run was started with the resulting conformers as input. (The molecule's hydrophobic tail for the induced fit docking (IFD) calculation was copied into an individual model from the structure generated by Ligprep.)

*Hotspot mapping with FTMap*

FTMap is a computational solvent mapping algorithm used to locate energetically robust binding regions (hotspots) on protein surfaces by placing multiple small organic probe molecules with different chemical properties on a dense grid around a protein (Brenke et al., 2009; Ngan et al., 2012). It then finds probe positions and orientations with low binding energies, first based on an empirical energy function, then by default it minimizes the 2000 lowest-energy positions for every probe based on the CHARMM potential with a continuum electrostatic term. It then clusters the positions of every probe type based on distance, and then clusters all of the probe clusters based on distance, resulting in so-called consensus clusters, which pinpoint the location of binding hotspots. The stronger the hotspot, the more consensus clusters it contains. One binding site can be composed of multiple nearby hotspots. In this study, we used FTMap in PPI mode.

*Ligand docking with Glide*

For ligand docking, Glide uses a series of hierarchical filtering steps to search for possible binding conformations of a given ligand (pose), with more and more accurate scoring of the selected poses (Friesner et al., 2004; Halgren et al., 2004). The properties of the receptor are represented on a pre-generated grid, by different sets of fields. By default, ligand flexibility is handled by a systematic rotamer generation before docking. The top-scored poses are submitted to a multistep energy minimization process. Finally, the best poses are scored with the Emodel scoring function and the best-scored pose is rescored with the Glide scoring function and retained.

Here, we used Glide in the SP precision, with default settings, in peptide docking mode, which retains more conformations during the docking process. For grid generation at Site1 and Site2, we set the center of the grid to be the center of the atomic coordinates of the consensus cluster representations at the given site. For Site3, we set the center of the grid to be the center of the atomic coordinates of the consensus cluster representation and the atomic coordinates of the β-OG ligand in the aligned structure of the 1OKE complex to the given structure with the "align" function of Pymol.

*Induced Fit Docking (IFD)*

To generate an appropriate binding pose in Site3, we selected the A chain of 5JHM as a starting structure, because it contains the highest-ranking consensus cluster near the cryptic site. We first opened the hairpin loop (274-286) by rebuilding it as a homology

model, using the open hairpin conformation in PDB entry 1OKE (269-281) as a template. Next, we docked the fluorinated tail of compound **7** into the open hydrophobic pocket with the Induced Fit Docking protocol of the Schrödinger suite (Sherman et al., 2006) (https://sciwheel.com/work/citation?ids=1608895&pre=&suf=&sa=0&dbf=0), with the grid center set to be the center of the atomic coordinates of the β-OG ligand in the aligned structure of the 1OKE complex to the open protein with Pymol align. Next, the 274-286 loop was refined using Prime with the docked ligand in place. Then, every side chain from 5.0 Å of the docked ligand was refined. Finally, the ligand was redocked to the refined protein structure with Glide, using the same settings as before. The poses were ranked according to the IFD score, which is the Glide score + 5% of the Prime energy of the protein. From the resulting complexes, we selected the one with the best IFD score and used its protein structure for docking calculations for the whole ligand.

*Prime MM/GBSA calculations*

Prime MM/GBSA implements a rescoring protocol for ligand–protein complexes for a more accurate ranking of ligands. The MM/GBSA $dG_{bind}$ score is calculated using the following expression:

$$dG_{bind} = E_{complex(minimized)} - E_{ligand(minimized)} - E_{protein(minimized)}$$

Here, the respective terms mean the Prime energies calculated for the minimized complex, ligand and protein. The energies are extended with a solvation term, calculated with the VSGB implicit solvation model (Li et al., 2011). Residues within a 5.0 Å distance from the ligand in the complex were treated as fully flexible, and the force field was set to OPLS4 (Lu et al., 2021).

*Molecular Dynamics simulations*

Molecular dynamics (MD) simulations were performed using the Desmond simulation suite to evaluate the stability of the determined binding poses, using the minimized protein–ligand complex acquired from the MM/GBSA calculations. Production runs of 100 ns were conducted on five replica systems with randomly sampled initial velocities for each pose, with the OPLS4 force field at 300K and atmospheric pressure. The simulations were set up using the default simulation protocol in Desmond, as follows (Bowers et al., 2006). The systems were set up with an orthorhombic simulation box with 10 Å padding, solvated with TIP4P explicit waters (Jorgensen et al., 1983) and neutralized by adding 22 Na+ ions. The salt concentration was set to 0.15 M by adding further Na+ and Cl- ions. The recording interval was set to 100 ps, resulting in 1000 recorded frames per simulation. The simulations were conducted according to the default Desmond settings using a 2 fs timestep RESPA integrator, Nosé–Hoover thermostat (Hoover, 1985) and Martyna–Tobias–Klein barostat (Martyna et al., 1994). Coulombic interactions were neglected between charges greater than 9.0 Å. The systems were subjected to the default Desmond relaxation protocol before the actual simulation. For each binding site, five parallel NPT simulations with random initial velocities were carried out for 100 ns with the OPLS4 force field (Lu et al., 2021) at 300K and atmospheric pressure.

The simulations were assessed by plotting the root mean squared distance (RMSD) values of the protein C$\alpha$, as well as the ligand heavy (non-hydrogen) atoms over time, after superimposing the protein onto the starting frame.

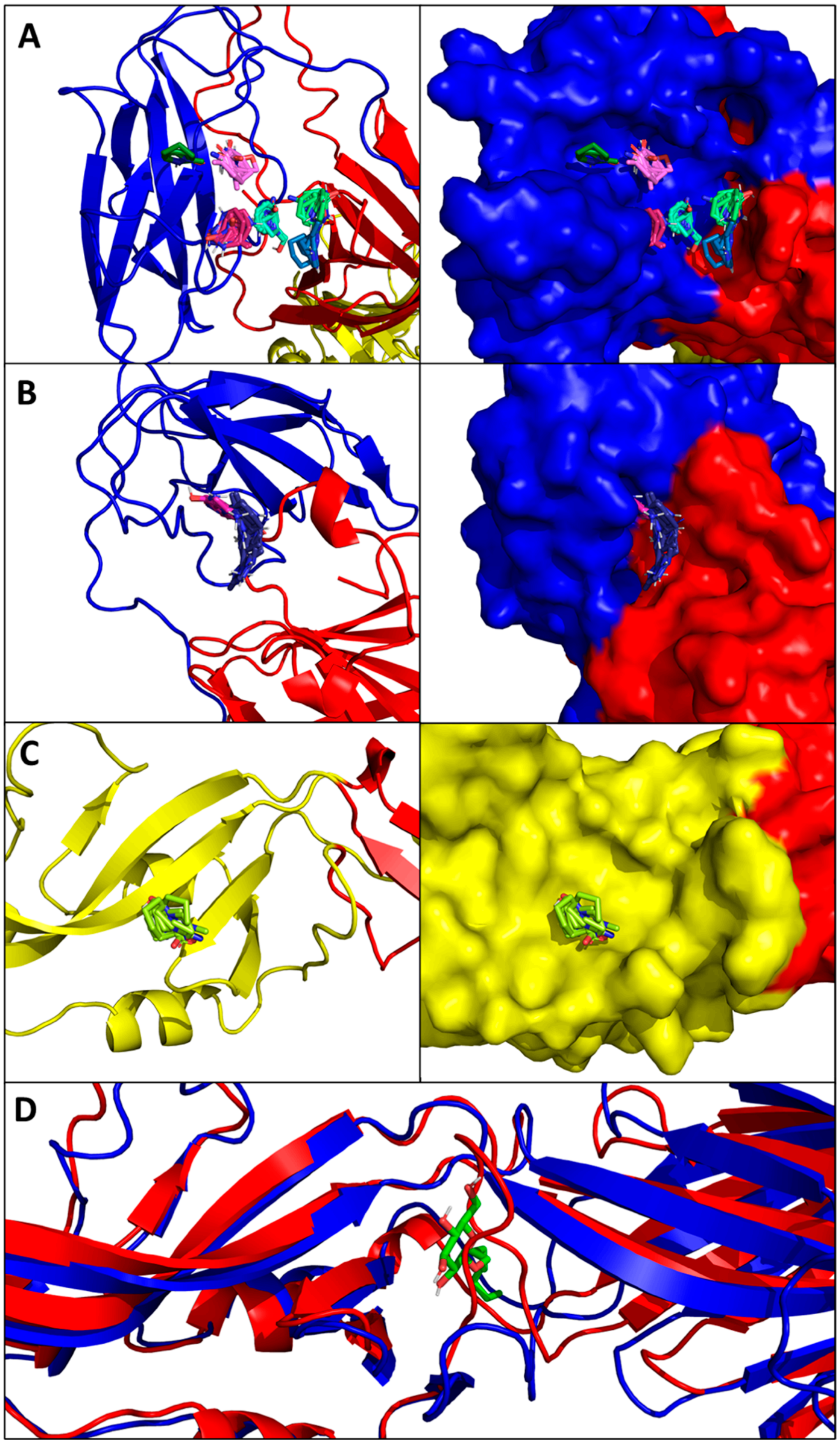


**Figure S1.** Binding sites identified with FTMap (A, B, C: Site1, Site2 and Site3 respectively), with surface representation on the left and cartoon representation on the right, colored according to the different domains (domain I: red, domain II: yellow, domain III: blue). Hotspots are represented with sticks for the centroids of probe clusters identified by FTMap. D) The structure of ZIKV E protein in the dimeric conformation (red, PDB ID: 5JHM), aligned with the structure of DENV E protein dimer (blue, PDB ID: 1OKE), complexed with n-octyl-β-D-glucoside (β-OG, green sticks). The open conformation of the hairpin loop observed in the dengue structure allows for the binding of a small molecule in this site.

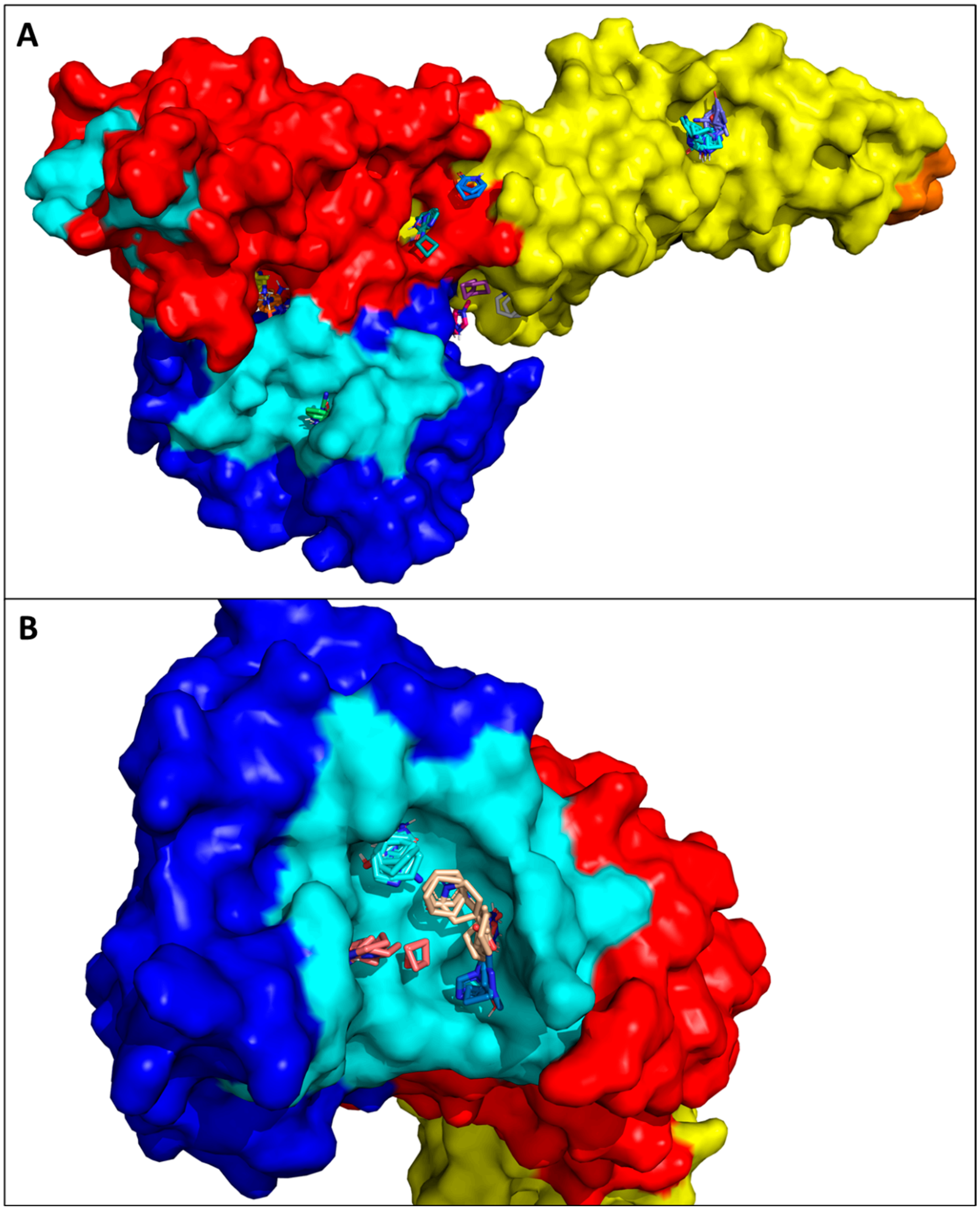


**Figure S2.** A) Binding sites on one chain from the trimeric structure of the dengue E protein (1OK8). The protein chain is shown in surface representation (red for domain I, yellow for domain II and blue for domain III), and the FTMap consensus clusters are represented with sticks, with a different color for each cluster. The residues corresponding to Site1 on the ZIKV E protein in its dimeric conformation are shown in cyan: clearly, Site1 disappears due to the structural reorganization during trimer formation. (The fusion loop is colored orange.) B) By comparison, Site1 is present in the dimer form of the DENV E protein (PDB ID: 1OAN), at the same location as in the ZIKV E protein.

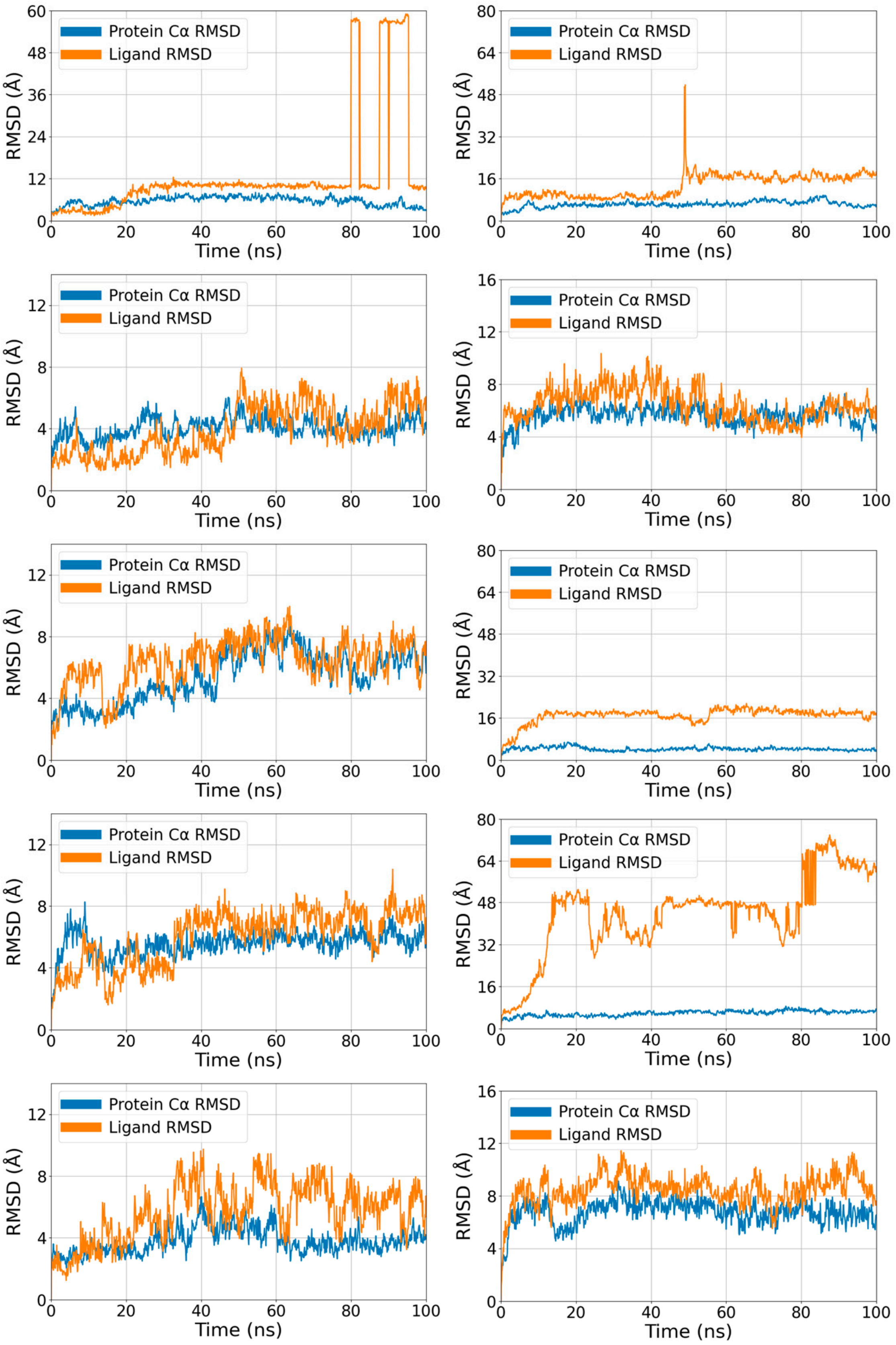
Protein Cα RMSD
Ligand RMSD
RMSD (Å)
Time (ns)

**Figure S3.** Root mean squared distance (RMSD) values over time for the protein C$\alpha$ and ligand heavy atoms through the five simulation replicas for Site1 (left) and Site2 (right). For Site1, both the protein and the ligand reach equilibrium in all simulations. (The anomaly in simulation 1 is due to the ligand traversing through the border of the periodic boundary box.) The relatively large ligand RMSD values (6-8 Å) are in line with its size and the flexibility of certain parts, particularly the perfluorinated tail and the glycan unit. For Site2, the ligand is observed to dissociate from the protein in three out of five simulations, as evidenced by RMSD values over 15 Å. (The high peak in simulation 1 and the multiple narrow peaks at the end of simulation 4 are, once again, caused by the ligand traversing through the periodic boundary box borders.)

**Table S1.** MM/GBSA dGbind (kcal/mol) scores at different sites for compound 7.

| **Compound 7** | | | |
|---|---|---|---|
| Site | Site1 | Site2 | Site3 |
| MM/GBSA dGbind | -76.87 | -39.81 | - |

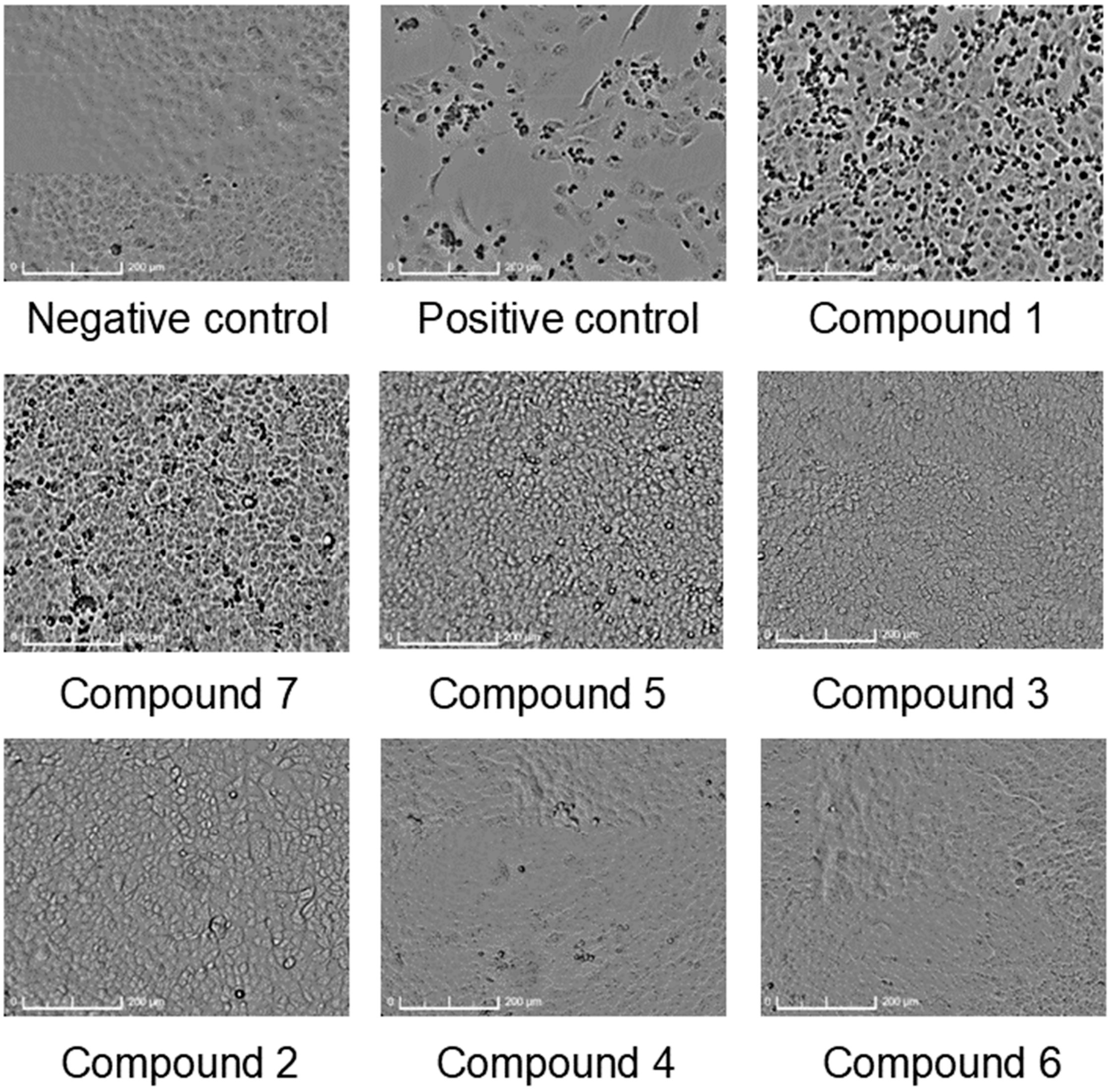


**Figure S4.** The Vero E6 cells were infected (MOI 1) and treated with the compound at MCC. We incubated them for 5 days. In the case of compounds 2, 3, 4 and 6 we did not observed any CPE. The pictures were taken by CytoSMART™ Omni.

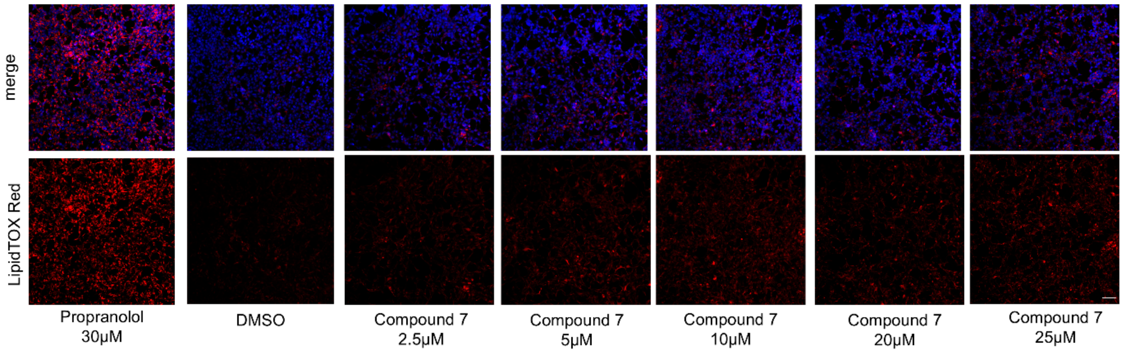

**Figure S5.** Phospholipidosis staining in Vero E6 cells following treatment with compound **7**. Vero E6 cells were treated with compound **7** at the indicated concentrations for 48 h. Propranolol (30 µM) was used as positive control and DMSO as vehicle control. Phospholipid accumulation was detected using LipidTOX™ Deep Red phospholipidosis stain (red), and nuclei were counterstained with Hoechst 33342 (blue). Representative confocal microscopy images are shown. Scale bar: 100 µm.

## Structures of the studied glycopeptide antibiotic derivatives

| Name/ number | Structure |
|---|---|
| Teicoplanin | 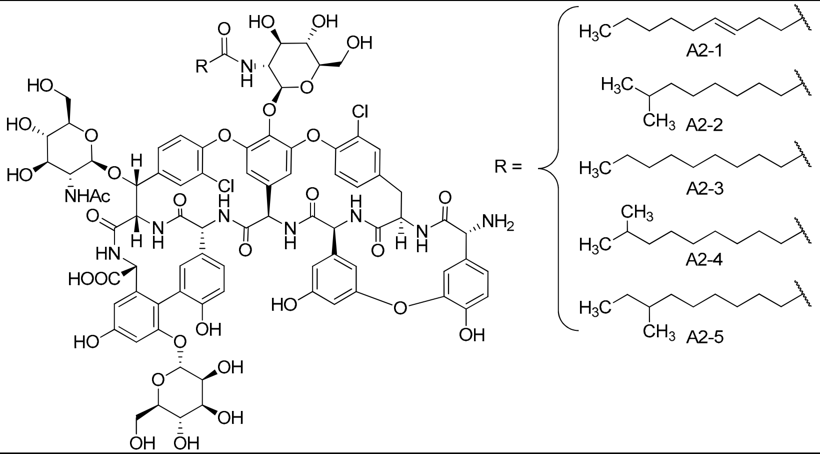 |
| Teicoplanin pseudoaglycone TC | 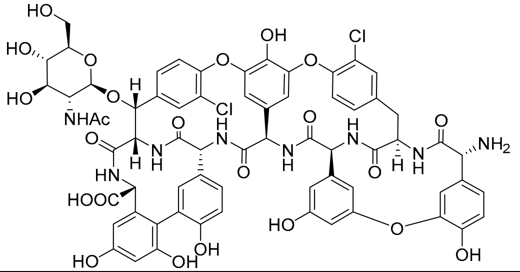 |
| Teicoplanin pseudoaglycone TB | 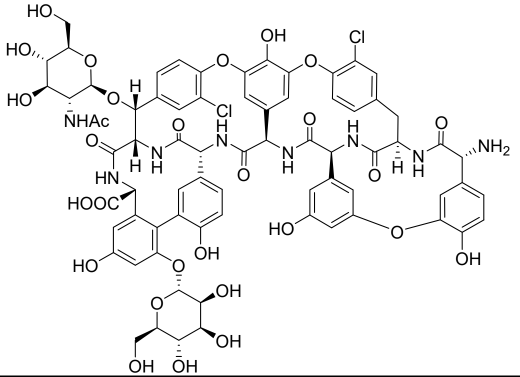 |

| | |
|---|---|
| Ristocetin | 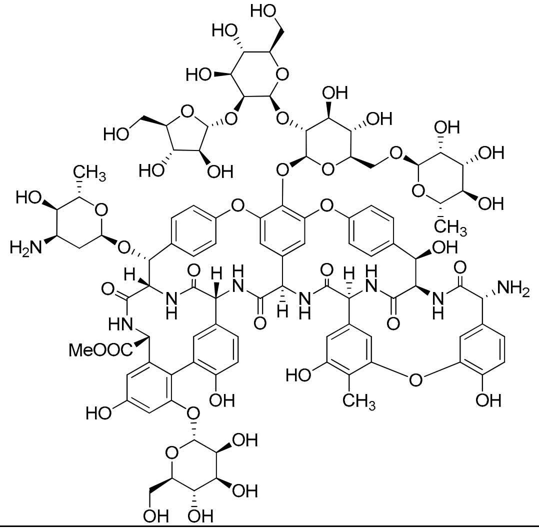 |
| Ristocetin aglycone | 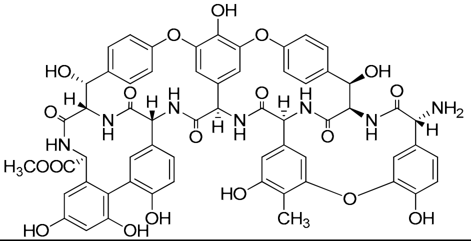 |
| Vancomycin | 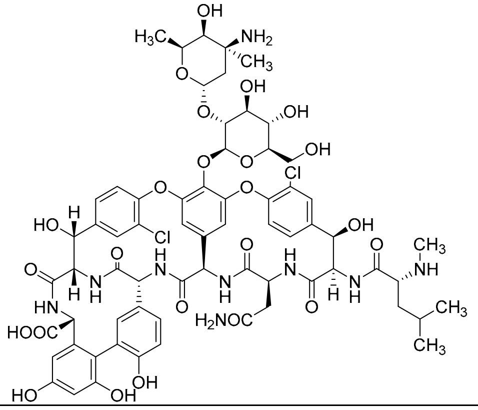 |
| **1** | 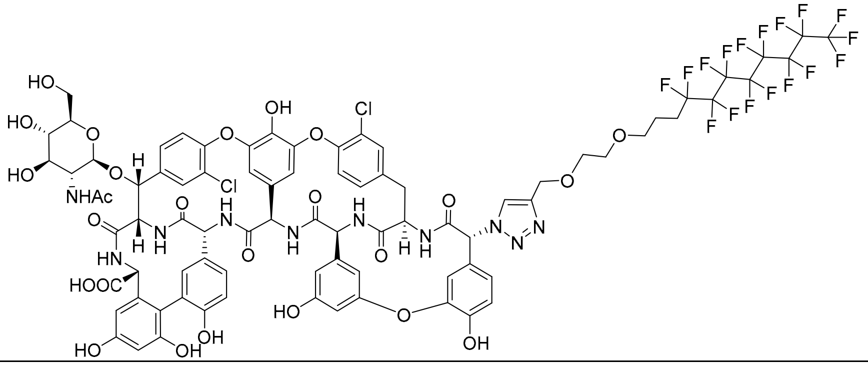 |

| | |
|---|---|
| **2** | 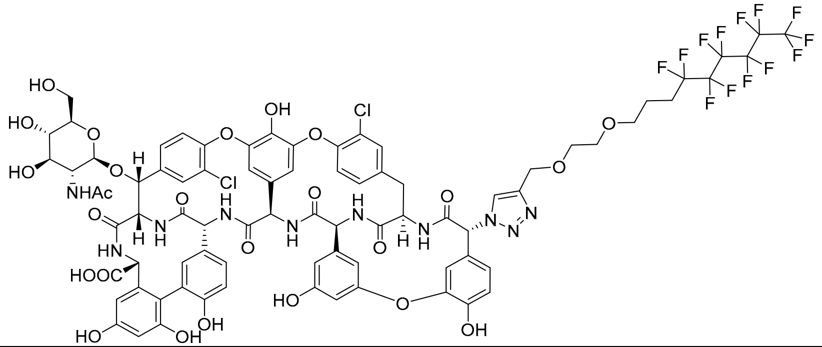 |
| **3** | 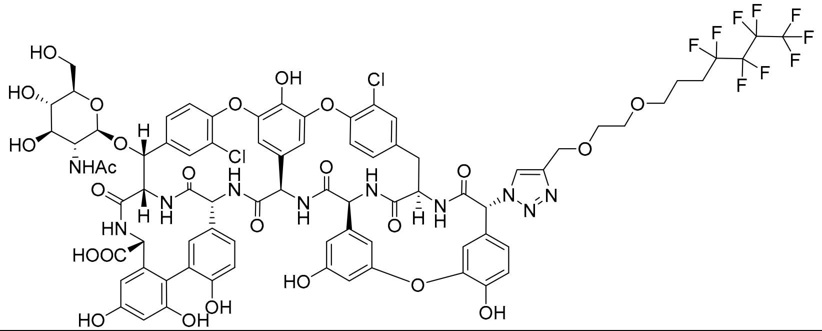 |
| **4** | 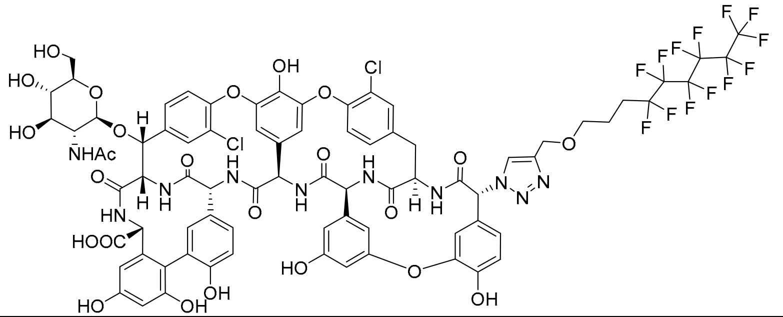 |
| **5** | 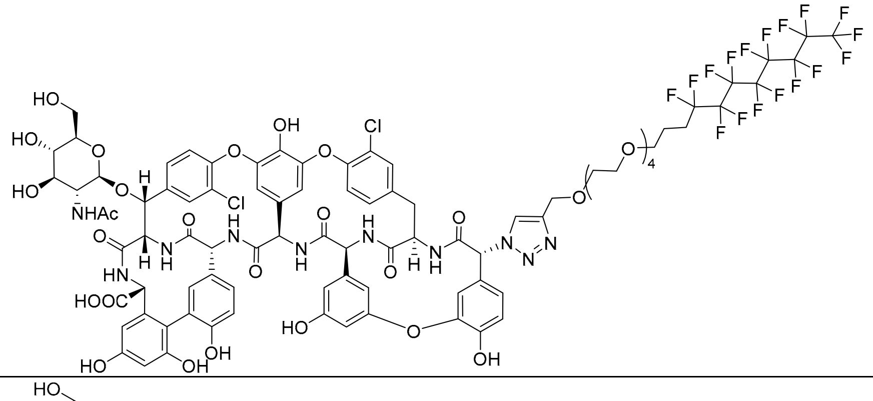 |
| **6** | 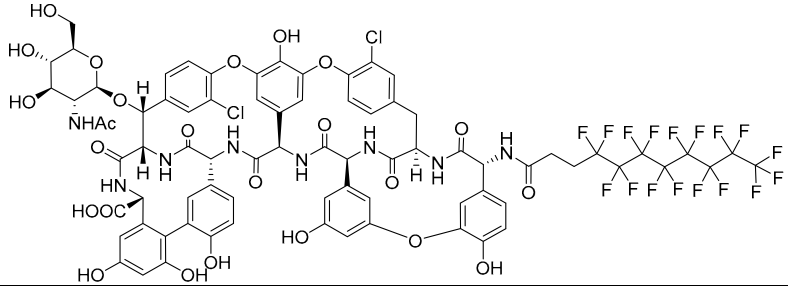 |

| | |
|---|---|
| **7** | 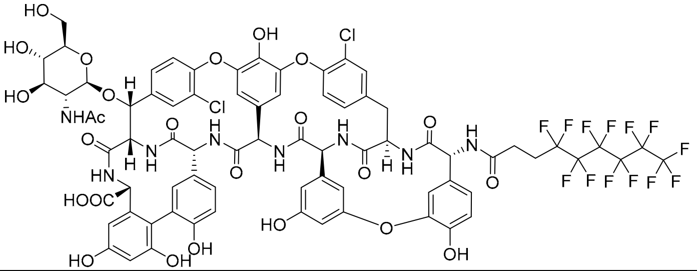 |
| **8** | 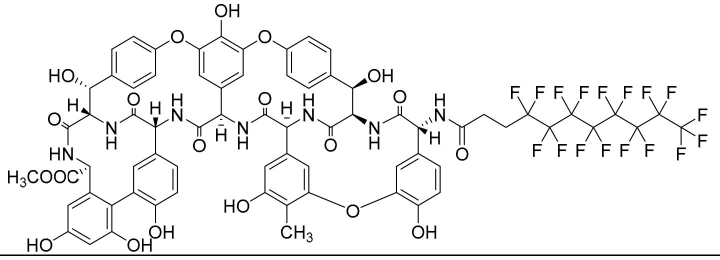 |

## NMR analysis of compounds 2, 7 and 8

Protonated carbons were assigned but quaternaries are only partially assigned because of signal overlaps. However, 1D $^{13}C$ NMR spectra are shown with peak lists.

*Compound 2*

Numbering of compound **2**

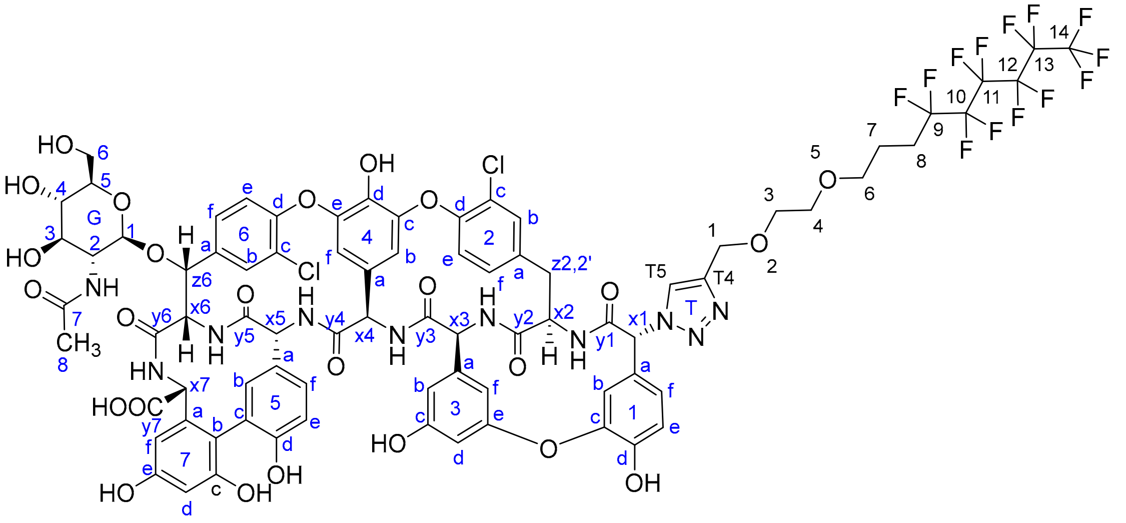

HSQC spectra of compound **2**

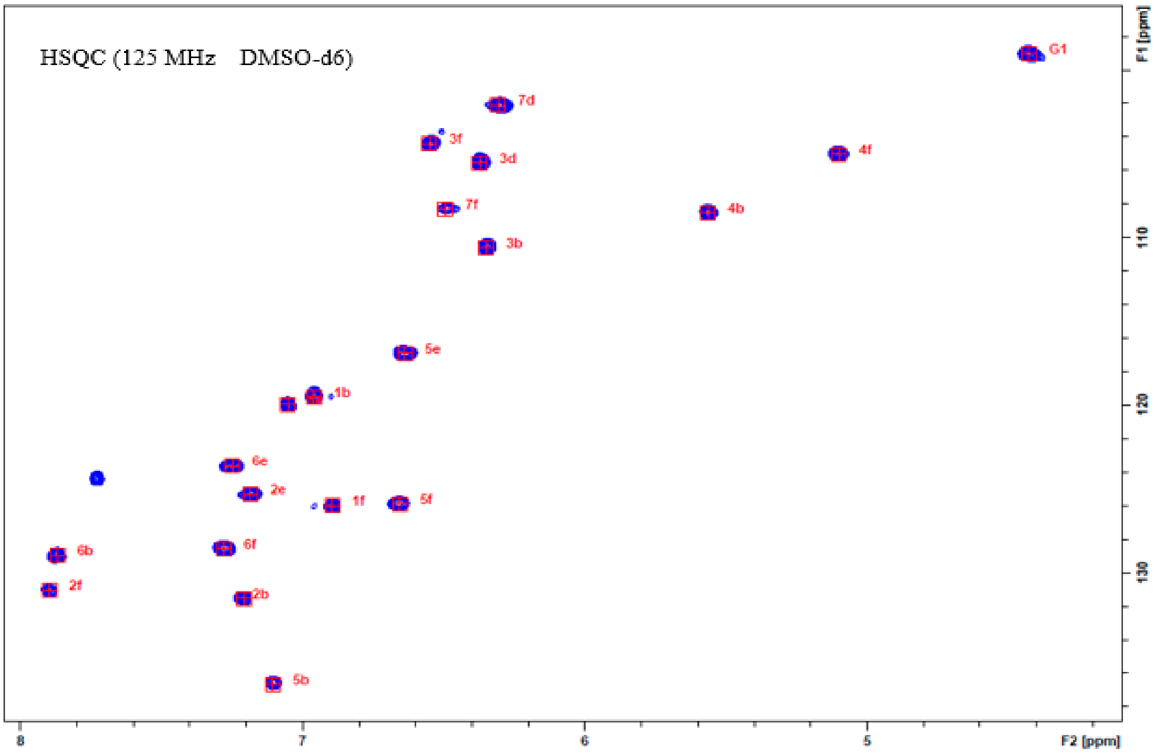

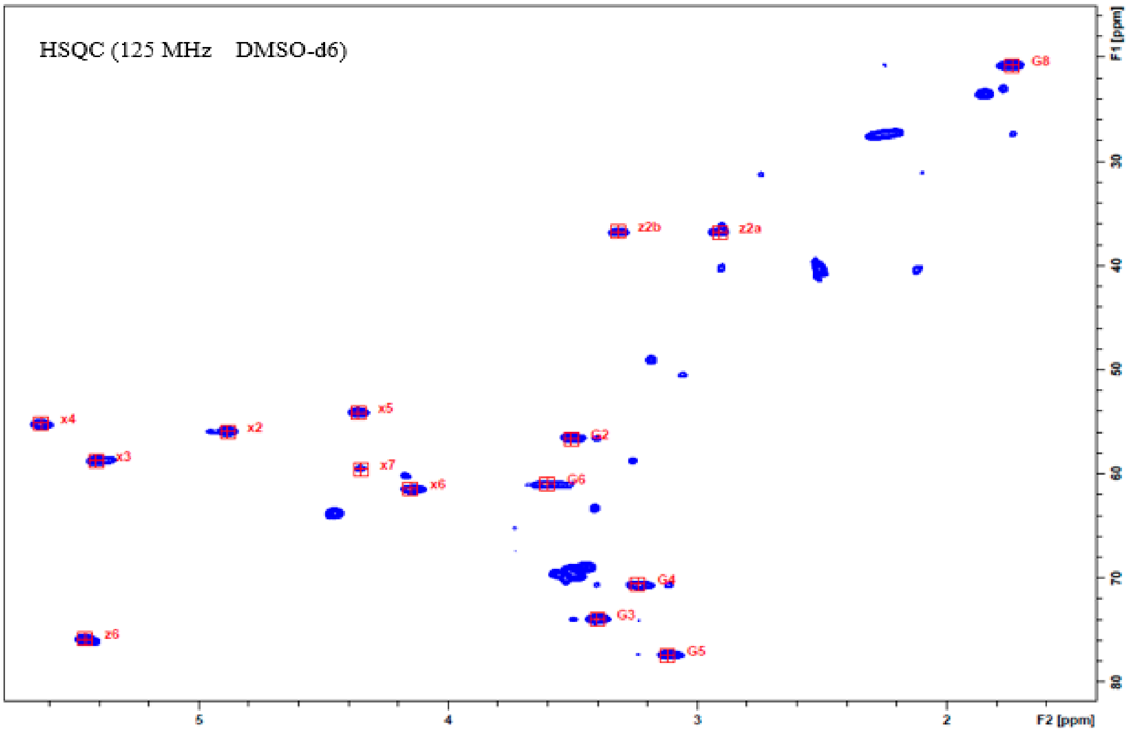


$^{1}H$-$^{13}C$ HSQC NMR assignment of compound **2**

| Compound 2 | | |
|---|---|---|
| **$^{1}H$ [ppm]** | **$^{13}C$ [ppm]** | **Annotation** |
| 7,11 | 136,64 | 5b |
| 7,21 | 131,51 | 2b |
| 7,87 | 128,95 | 6b |
| 7,28 | 128,54 | 6f |
| 6,90 | 125,99 | 1f |
| 6,66 | 125,88 | 5f |
| 7,18 | 125,32 | 2e |
| 7,90 | 131,01 | 2f |
| 7,25 | 123,62 | 6e |
| 7,05 | 119,95 | 1e |
| 6,96 | 119,47 | 1b |
| 6,64 | 116,88 | 5e |
| 6,35 | 110,57 | 3b |
| 5,56 | 108,51 | 4b |
| 6,49 | 108,29 | 7f |
| 6,37 | 105,51 | 3d |
| 5,10 | 105,00 | 4f |
| 6,55 | 104,36 | 3f |
| 6,31 | 102,08 | 7d |
| 4,43 | 99,00 | G1 |
| 3,12 | 77,38 | G5 |
| 5,46 | 75,83 | z6 |
| 3,40 | 73,94 | G3 |
| 3,24 | 70,55 | G4 |
| 4,15 | 61,40 | x6 |
| 3,60 | 60,93 | G6 |
| 4,35 | 59,51 | x7 |
| 5,41 | 58,72 | x3 |
| 3,51 | 56,63 | G2 |

| | | |
|---|---|---|
| 4,89 | 55,93 | x2 |
| 5,63 | 55,13 | x4 |
| 4,36 | 54,14 | x5 |
| 2,91 | 36,77 | z2a |
| 3,32 | 36,71 | z2b |
| 1,74 | 20,74 | G8 |

*Compound 7*

Numbering of compound **7**

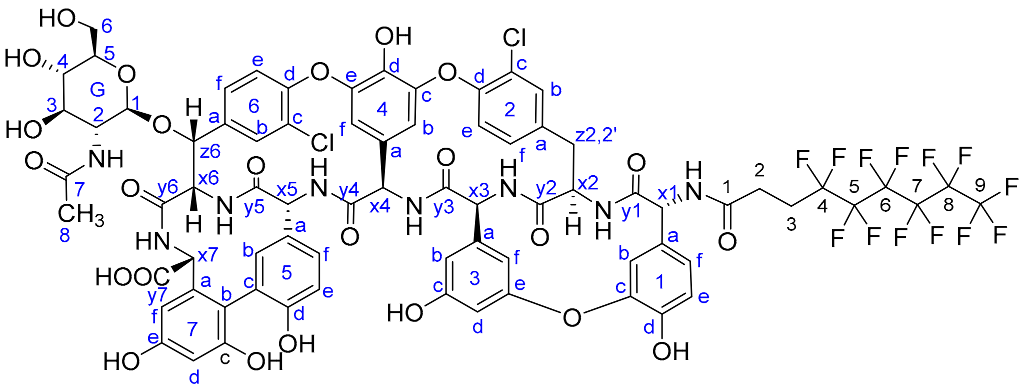

$^{19}$F NMR spectrum of compound **7**

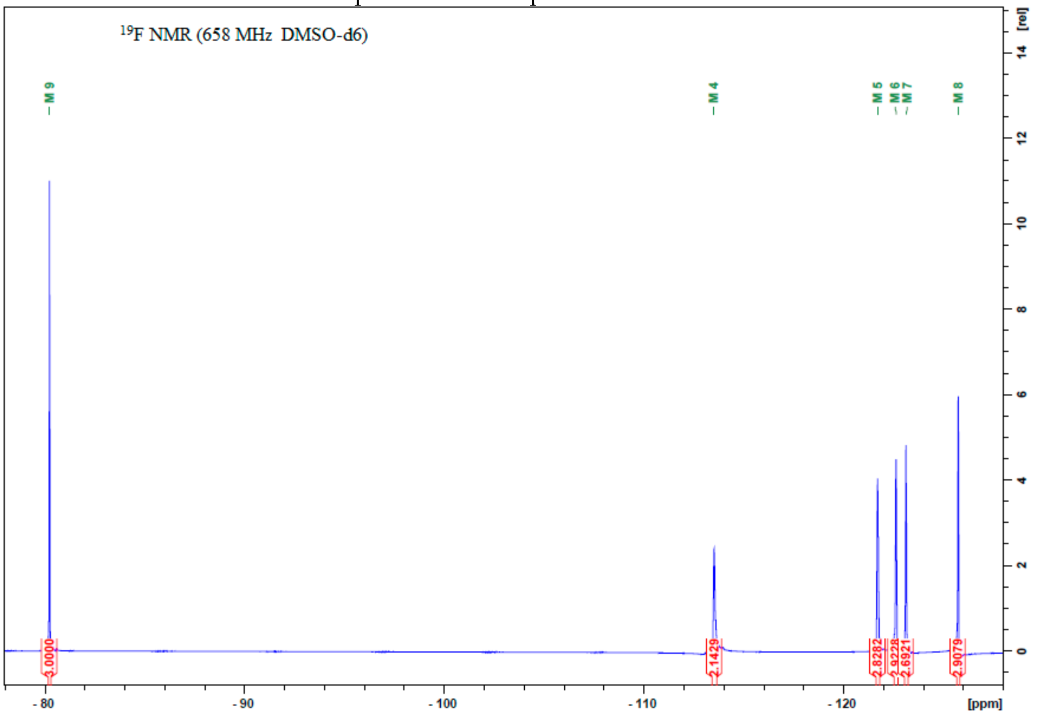


$^{19}$F NMR assignment of compound **7**

| Compound 7 | |
|---|---|
| **$^{19}$F [ppm]** | **Annotation** |
| -80.22 | 9 |
| -113.50 | 4 |
| -121.71 | 5 |
| -122.63 | 6 |

| | |
|---|---|
| -123.13 | 7 |
| -125.75 | 8 |

HSQC spectra of compound **7**

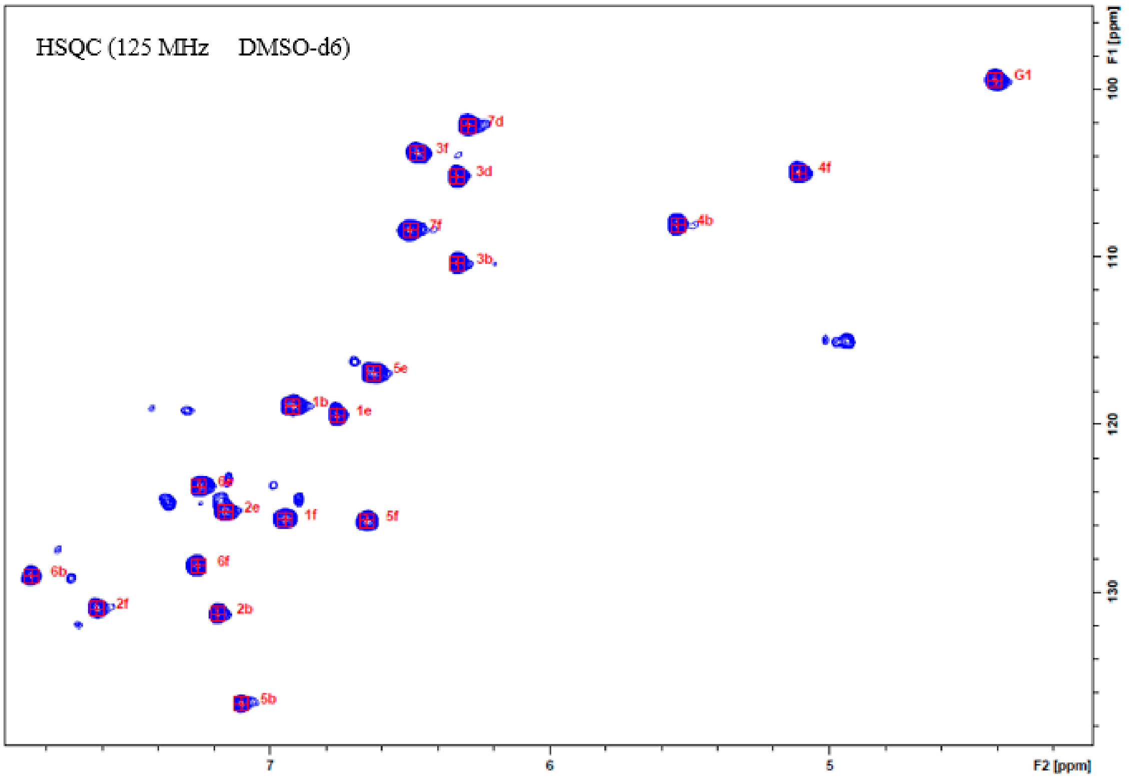

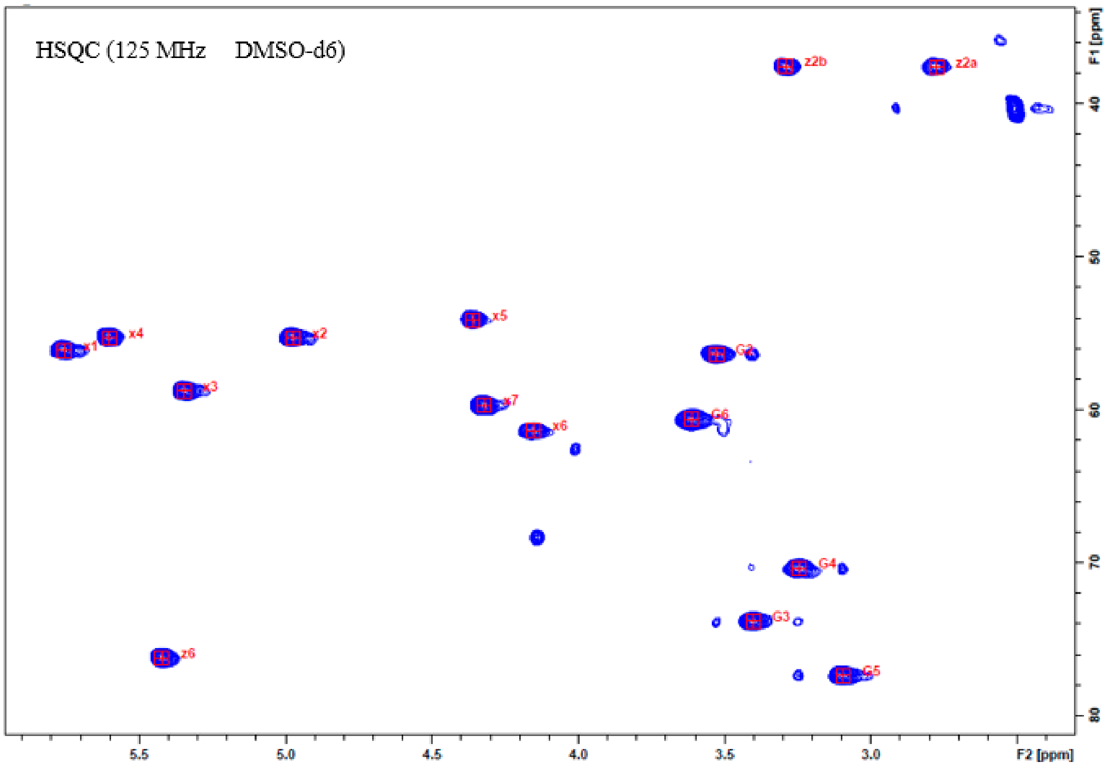


$^{13}$C spectra of compound **7**

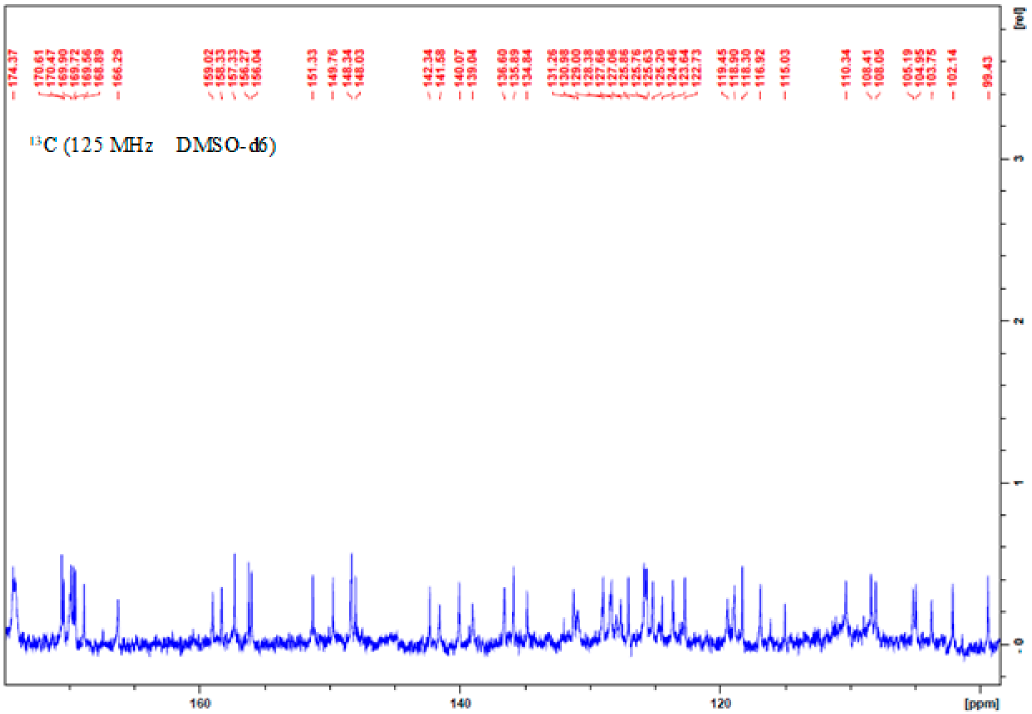

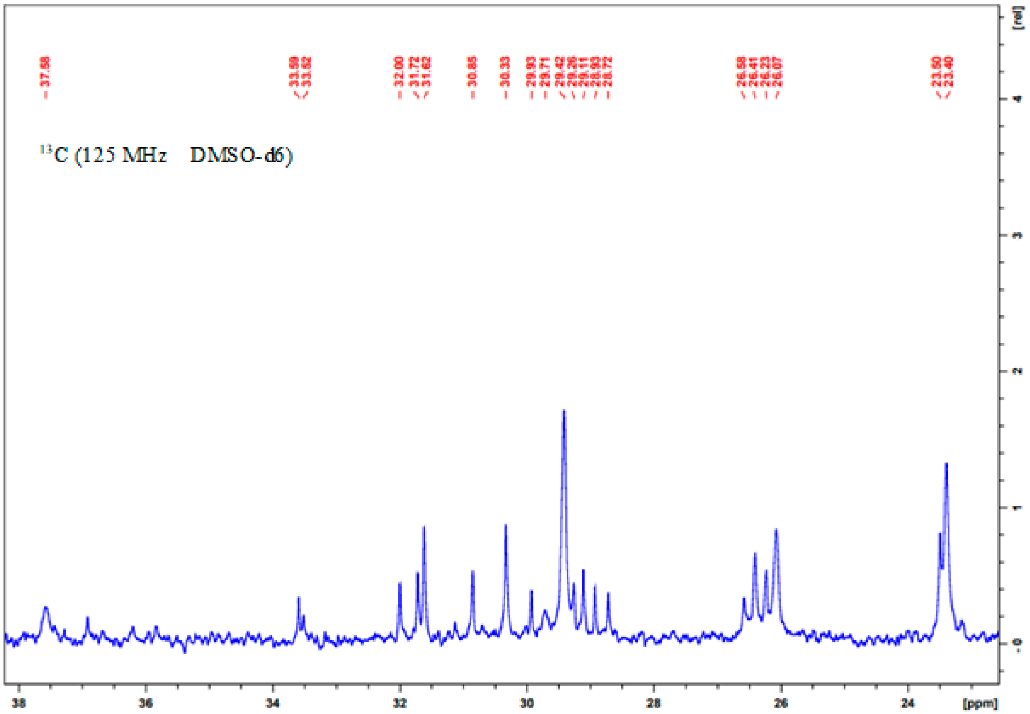

$^{1}H$-$^{13}C$ HSQC NMR assignment of compound **7**

| | **Compound 7** | |
|---|---|---|
| **$^{1}H$ [ppm]** | **$^{13}C$ [ppm]** | **Annotation** |
| 7,10 | 136,64 | 5b |
| 7,19 | 131,27 | 2b |
| 7,62 | 130,97 | 2f |
| 7,85 | 129,02 | 6b |
| 7,26 | 128,41 | 6f |
| 6,66 | 125,72 | 5f |
| 6,95 | 125,62 | 1f |
| 7,16 | 125,17 | 2e |
| 7,26 | 123,68 | 6e |
| 6,76 | 119,44 | 1e |
| 6,92 | 118,87 | 1b |
| 6,63 | 116,90 | 5e |
| 6,33 | 110,34 | 3b |
| 6,50 | 108,39 | 7f |
| 5,54 | 108,05 | 4b |
| 6,33 | 105,20 | 3d |
| 5,11 | 104,96 | 4f |
| 6,48 | 103,76 | 3f |
| 6,29 | 102,15 | 7d |
| 4,41 | 99,44 | G1 |
| 3,10 | 77,35 | G5 |
| 5,42 | 76,21 | z6 |
| 3,40 | 73,79 | G3 |
| 3,24 | 70,37 | G4 |
| 4,15 | 61,35 | x6 |
| 3,61 | 60,62 | G6 |
| 4,32 | 59,70 | x7 |
| 5,35 | 58,75 | x3 |
| 3,53 | 56,34 | G2 |
| 5,76 | 56,08 | x1 |

| | | |
|---|---|---|
| 4,97 | 55,26 | x2 |
| 5,60 | 55,26 | x4 |
| 4,36 | 54,11 | x5 |
| 2,78 | 37,56 | z2a |
| 3,29 | 37,52 | z2b |
| - | 169,56 | G2(C=O) |
| - | 174,37 | y7 |
| 1,26 | 22,44 | G2(CH3) |

*Compound 8*

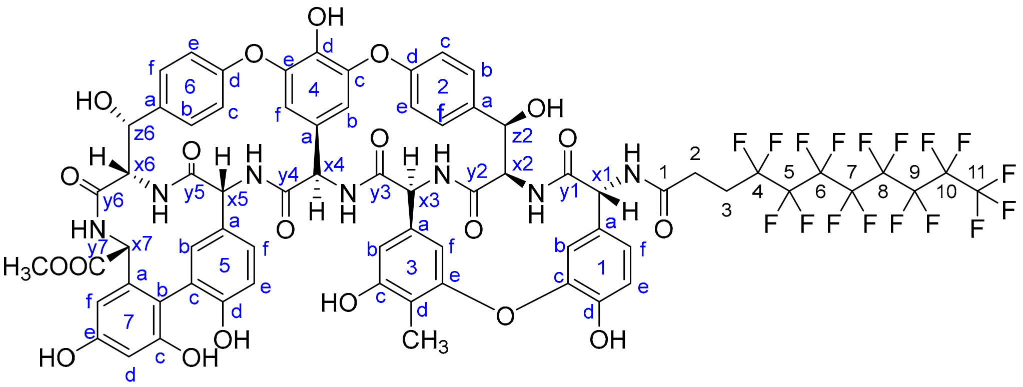

$^{19}$F NMR spectrum of compound **8.**

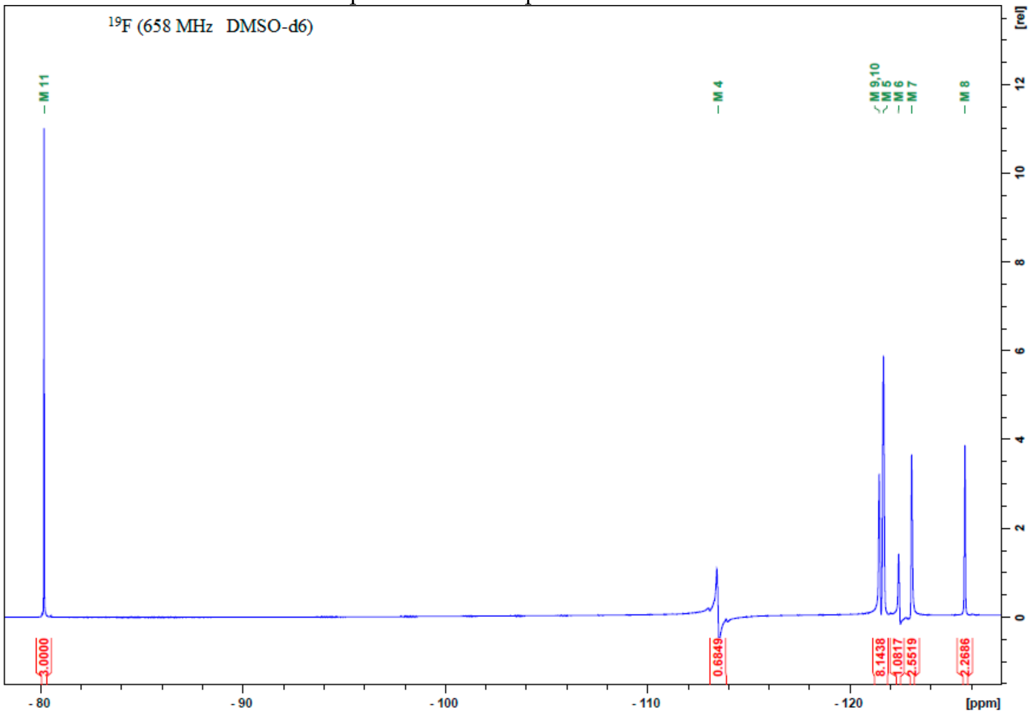


$^{19}$F NMR assignment of compound **8**

| **Compound 8** | |
|---|---|
| **$^{19}$F [ppm]** | **Annotation** |
| -80.17 | 11 |
| -113.49 | 4 |
| -121.45 | 9, 10 |

| | |
|---|---|
| -121.64 | 5 |
| -122.40 | 6 |
| -123.06 | 7 |
| -125.68 | 8 |

HSQC spectra of compound **8**

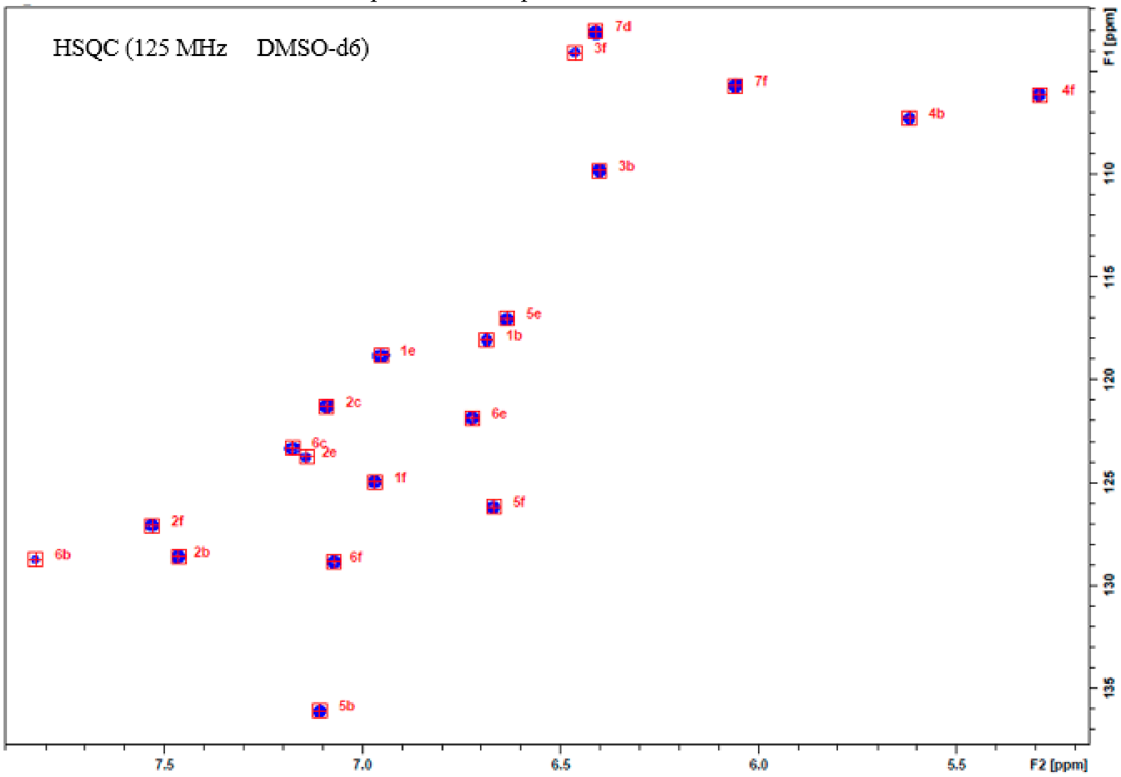

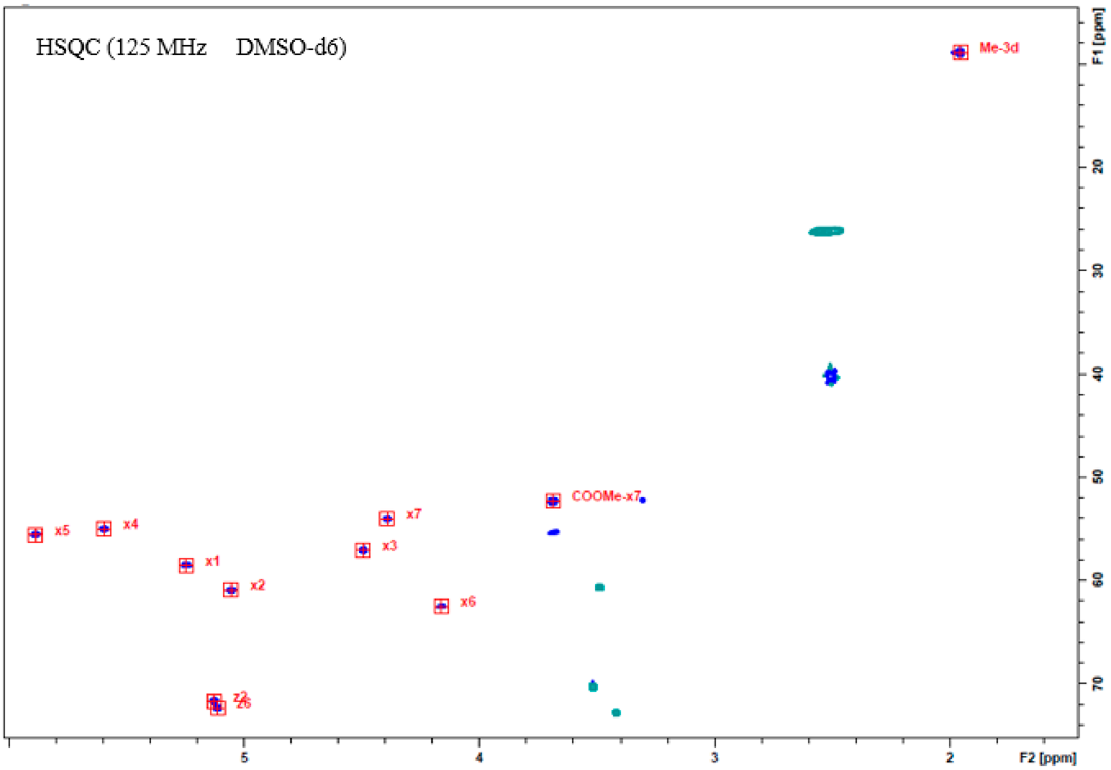


$^{13}$C spectra of compound **8**

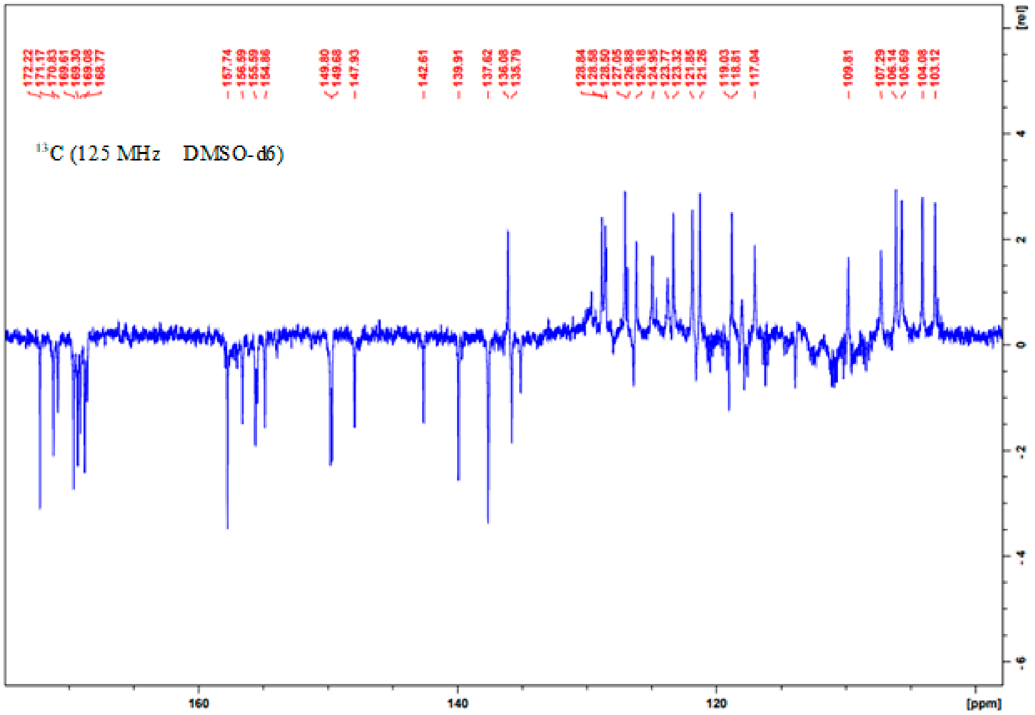

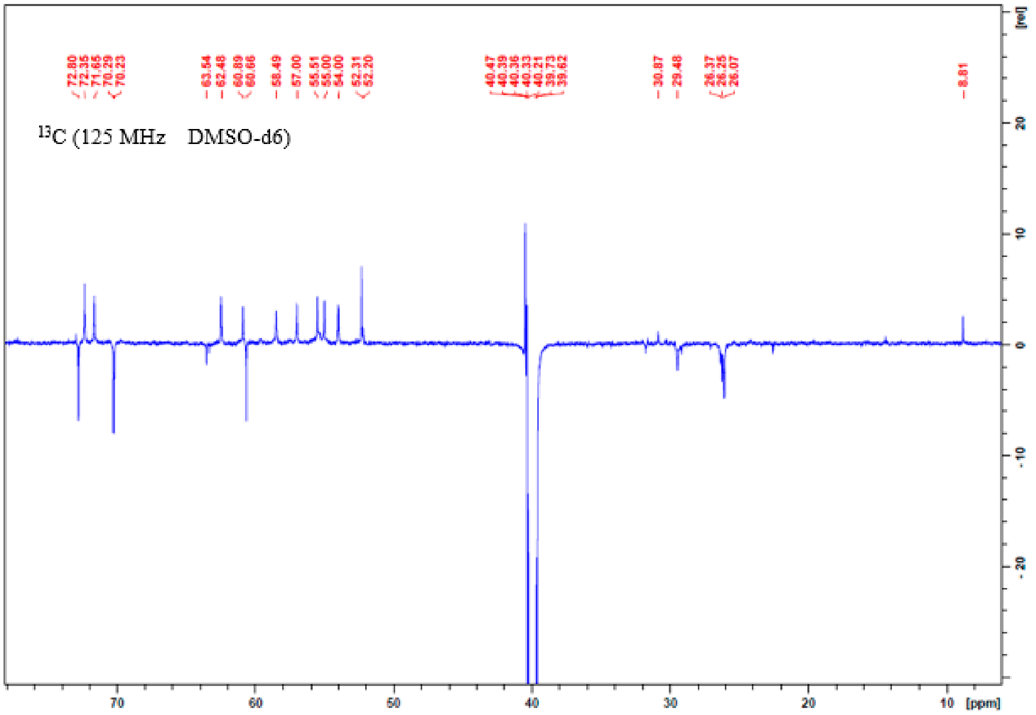


$^{1}H$-$^{13}C$ HSQC and $^{1}H$-$^{1}H$ HMBC NMR assignment of compound **8**

| Compound 8 | | |
|---|---|---|
| **$^{1}H$ [ppm]** | **$^{13}C$ [ppm]** | **Annotation** |
| 7,11 | 136,09 | 5b |
| 7,07 | 128,83 | 6f |
| 7,83 | 128,73 | 6b |
| 7,46 | 128,57 | 2b |
| 7,53 | 127,08 | 2f |
| 6,67 | 126,18 | 5f |
| 6,97 | 124,97 | 1f |
| 7,14 | 123,70 | 6e |
| 7,18 | 123,32 | 6c |
| 6,72 | 121,86 | 2e |
| 7,09 | 121,28 | 2c |
| 6,95 | 118,79 | 1e |
| 6,69 | 118,05 | 1b |
| 6,63 | 117,03 | 5e |
| 6,40 | 109,81 | 3b |
| 5,62 | 107,28 | 4b |
| 5,29 | 106,14 | 4f |
| 6,06 | 105,69 | 7f |
| 6,46 | 104,11 | 3f |
| 6,41 | 103,06 | 7d |
| 5,11 | 72,35 | z6 |
| 5,13 | 71,66 | z2 |
| 4,16 | 62,50 | x6 |
| 5,05 | 60,88 | x2 |
| 5,25 | 58,51 | x1 |
| 4,49 | 57,00 | x7 |
| 5,89 | 55,51 | x5 |
| 5,59 | 55,00 | x4 |
| 4,39 | 54,01 | x3 |

| | | |
|---|---|---|
| 3,69 | 52,32 | COOMe-x7 |
| 1,96 | 8,83 | Me-3d |
| 2,49 | 26,07 | 3 |
| 2,53 | 26,25 | 2 |
| - | 113,91 | 3d |
| - | 118,26 | 7b |
| - | 139,92 | 6a |
| - | 135,11 | 4d |
| - | 143,01 | 7a |
| - | 149,65 | 4c |
| - | 149,80 | 4e |
| - | 155,59 | 6d |
| - | 155,45 | 3e |
| - | 157,75 | 7c |
| - | 156,59 | 3c |
| - | 157,75 | 7e |
| - | 169,31 | 1 |
| - | 168,77 | y6 |
| - | 169,08 | y2 |
| - | 169,61 | y5 |
| - | 170,83 | y3 |
| - | 172,22 | y7 |
| - | 171,17 | y4 |
| 5,11 | - | NH-6 |

## NMR spectra of the synthetic intermediates

*Compound 10*

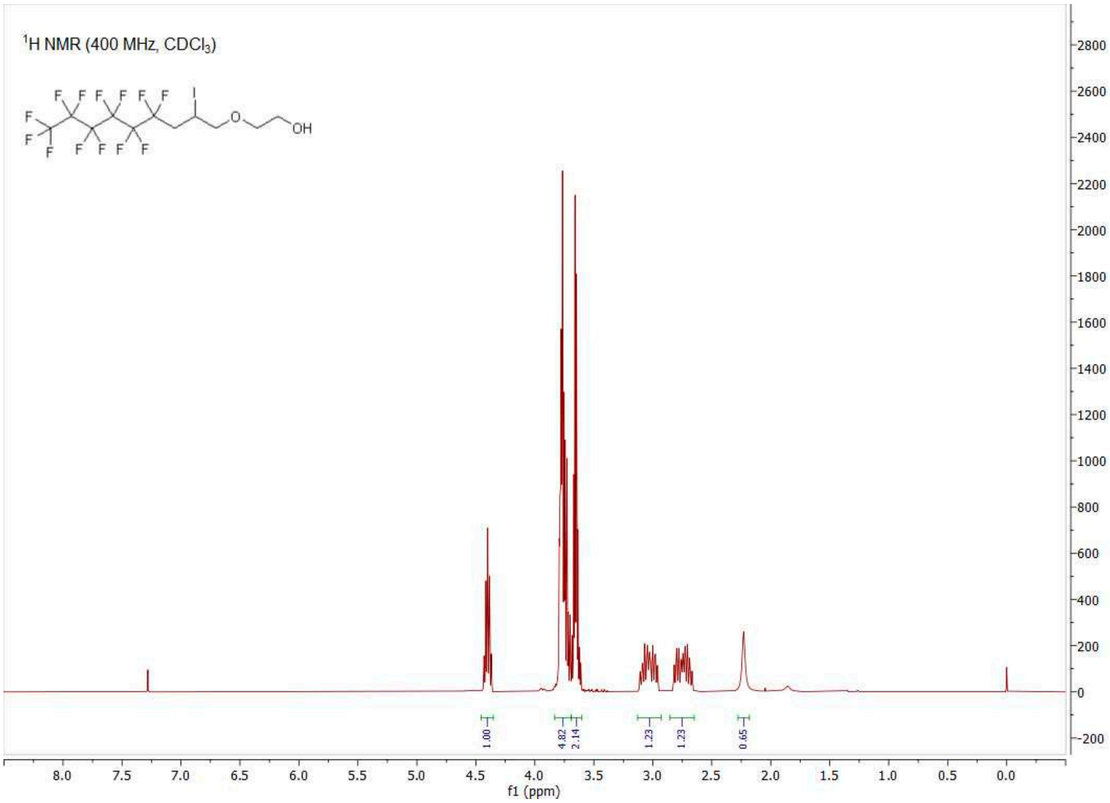

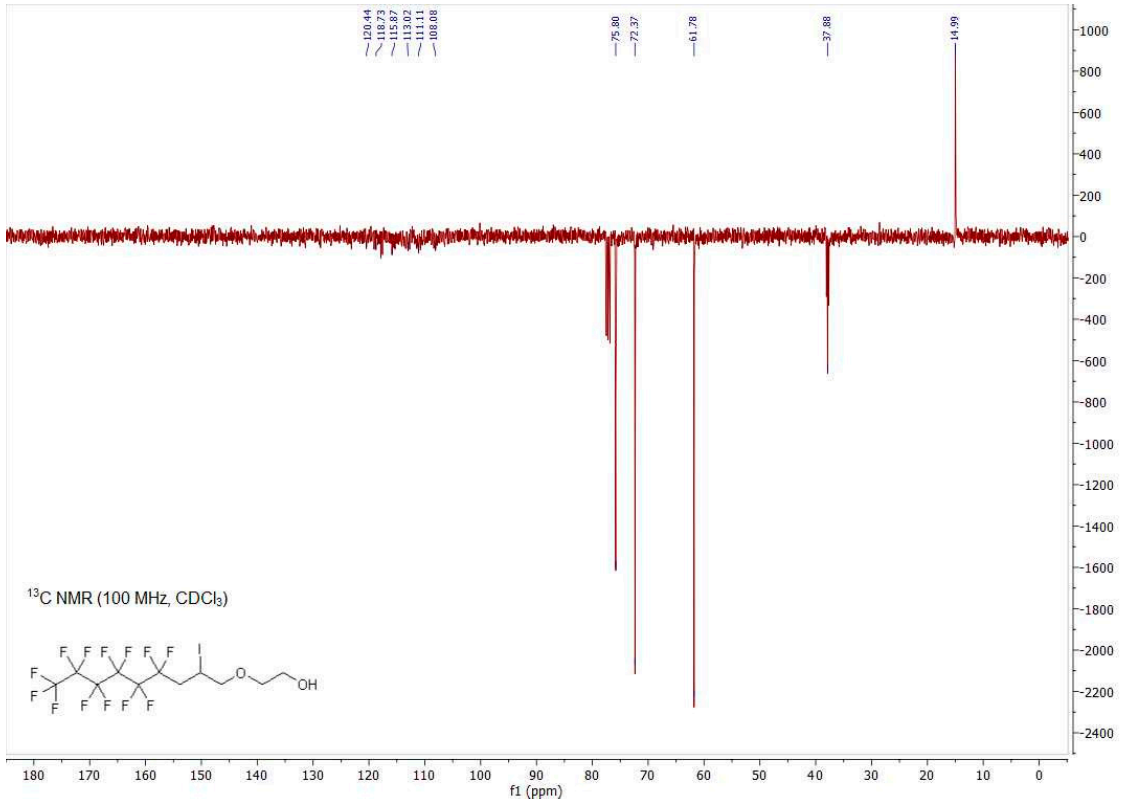

$^{13}C$ NMR (100 MHz, CDCl$_3$)
120.44
118.73
115.87
113.02
111.11
108.08
75.80
72.37
61.78
37.88
14.99
f1 (ppm)


*Compound 11*

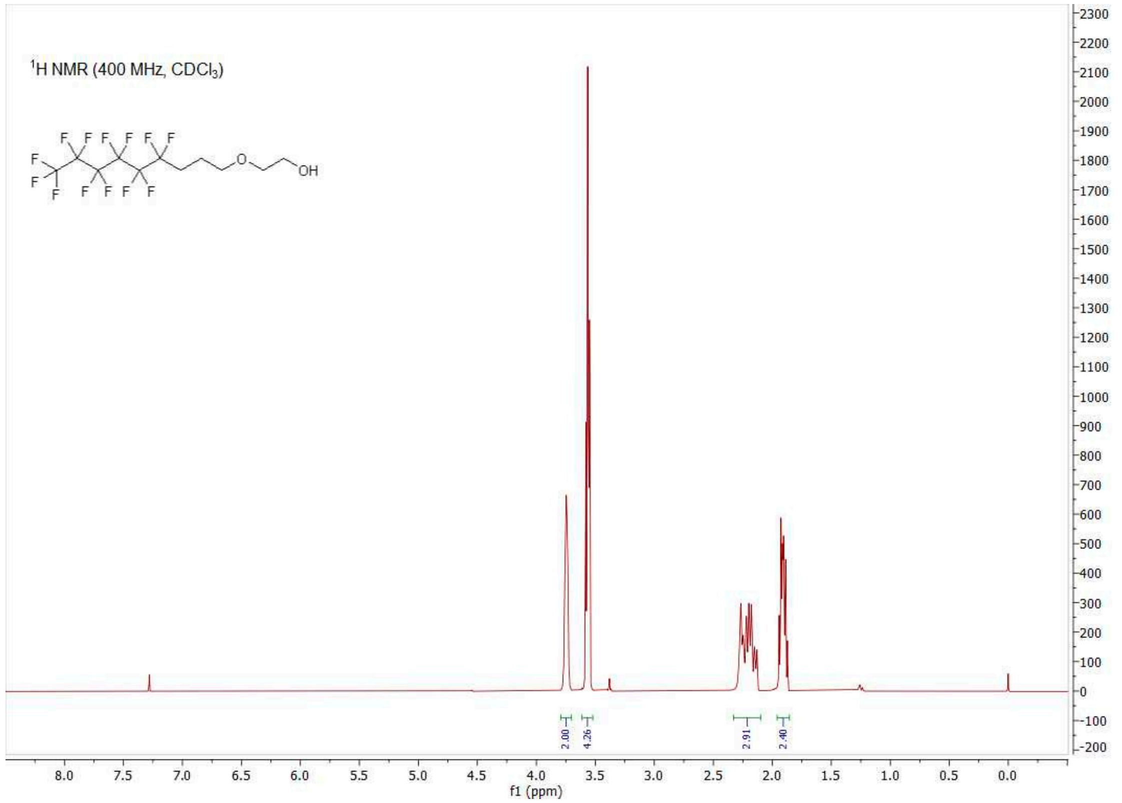

$^{1}H$ NMR (400 MHz, CDCl$_3$)
2.00
4.26
2.91
2.40
f1 (ppm)

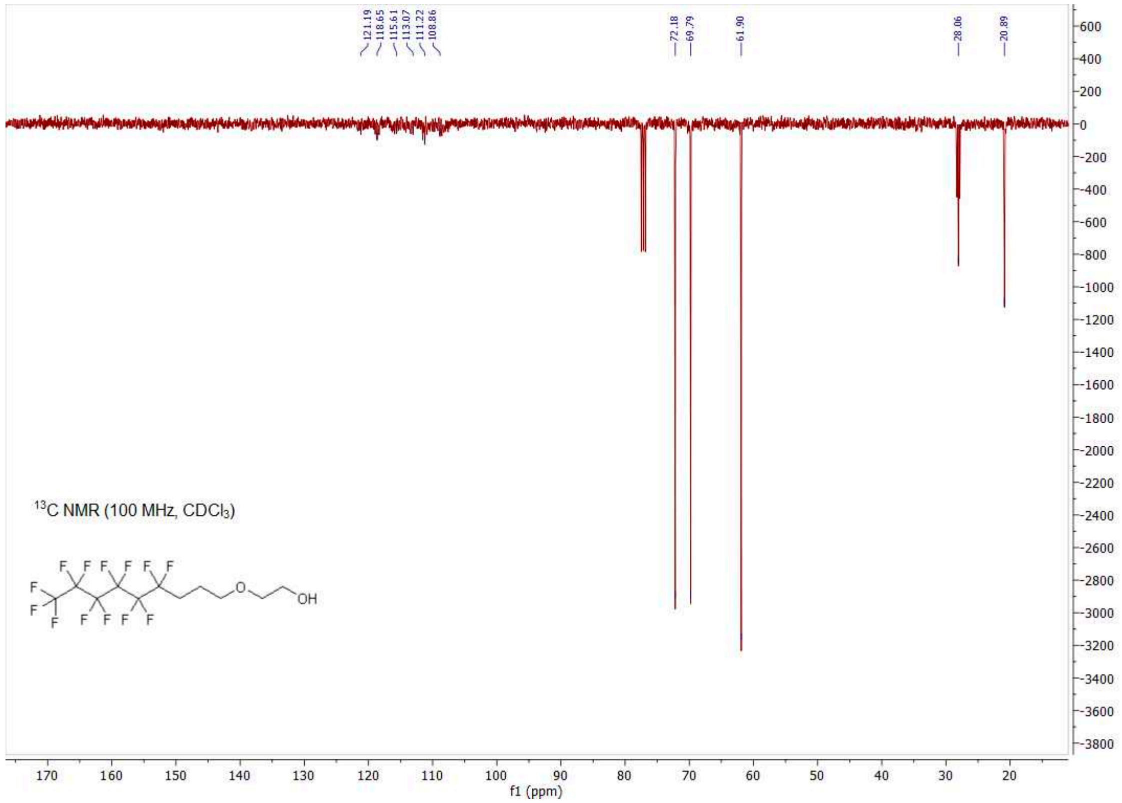

121.19
118.65
115.61
113.07
111.22
108.86
72.18
69.79
61.90
28.06
20.89
13C NMR (100 MHz, CDCl3)
OH
f1 (ppm)


*Compound 12*

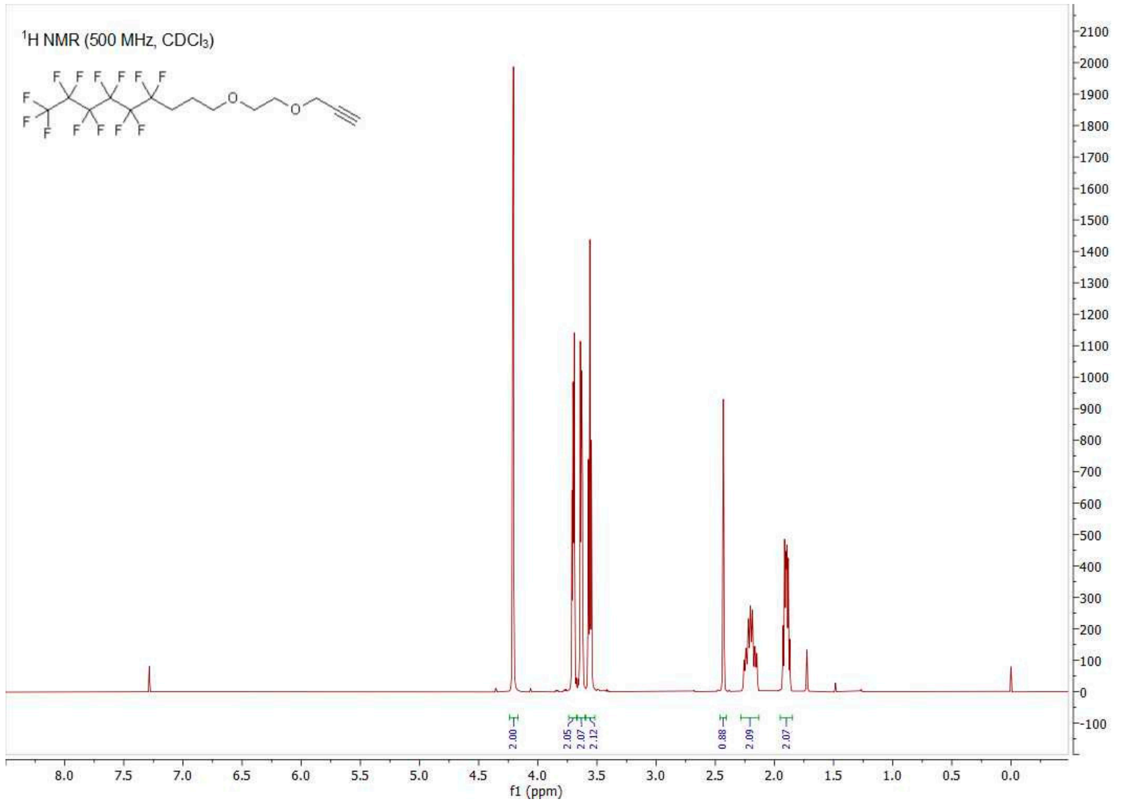

1H NMR (500 MHz, CDCl3)
2.00
2.05
2.07
2.12
0.88
2.09
2.07
f1 (ppm)

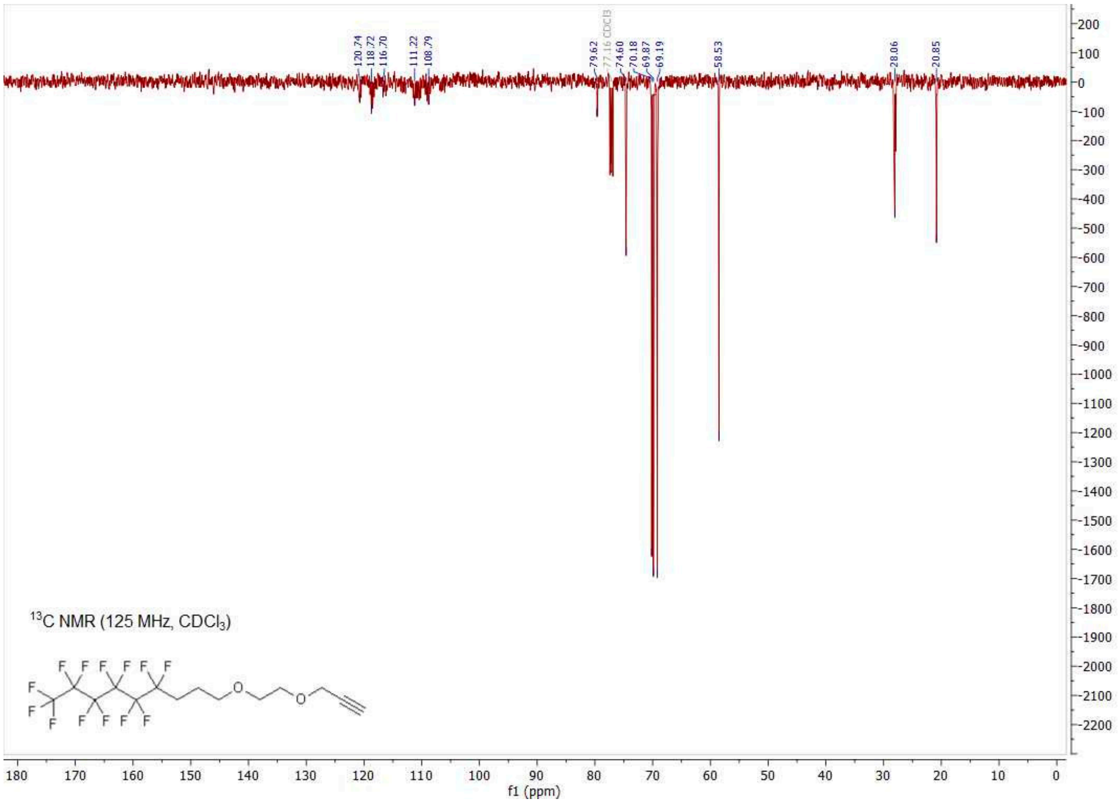
120.74
118.72
116.70
111.22
108.79
79.62
77.16 CDCl3
74.60
70.18
69.87
69.19
58.53
28.06
20.85
$^{13}$C NMR (125 MHz, CDCl$_3$)
f1 (ppm)

*Compound 15*

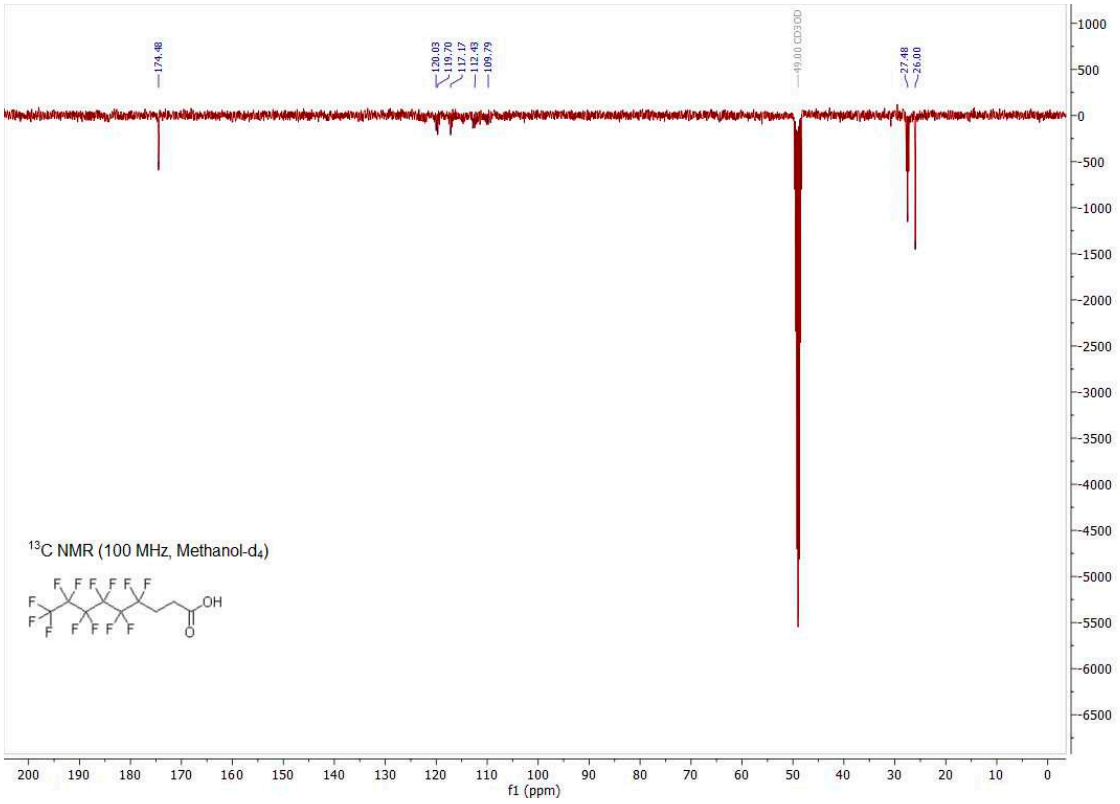
174.48
120.03
119.70
117.17
112.43
109.79
49.00 CD3OD
27.48
26.00
$^{13}$C NMR (100 MHz, Methanol-d$_4$)
f1 (ppm)

*Compound 16*

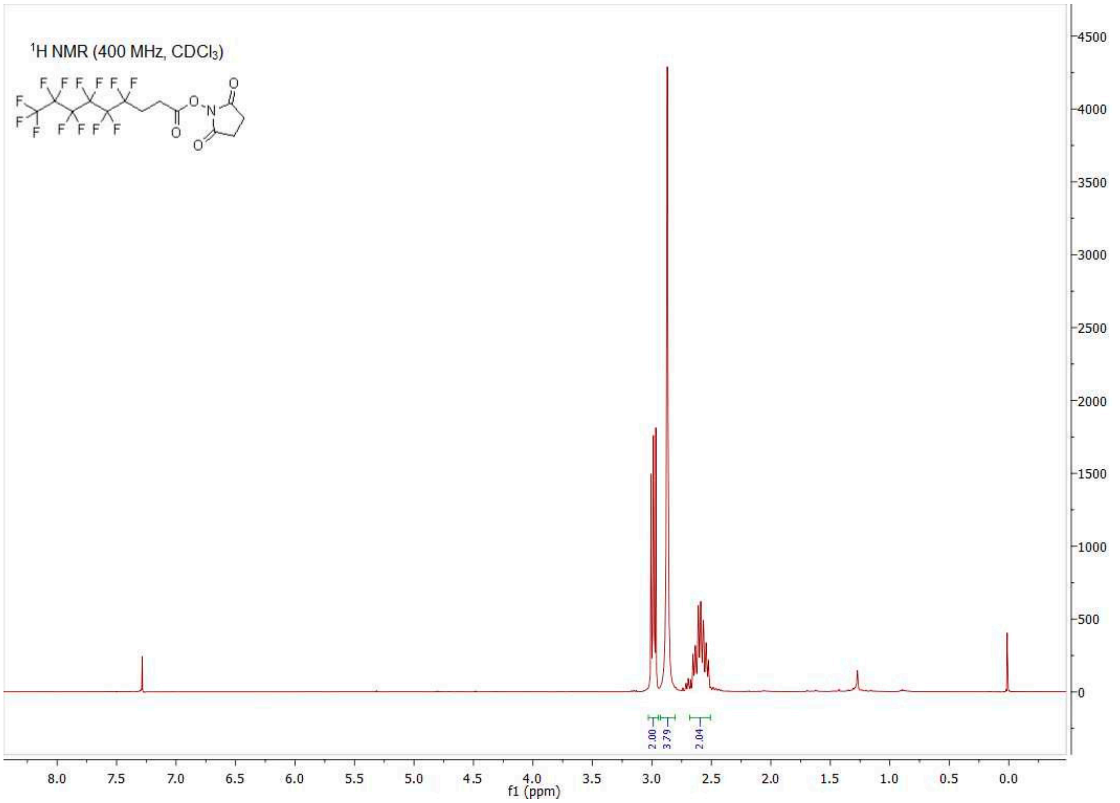

$^1$H NMR (400 MHz, CDCl$_3$)
2.00
3.79
2.04
f1 (ppm)


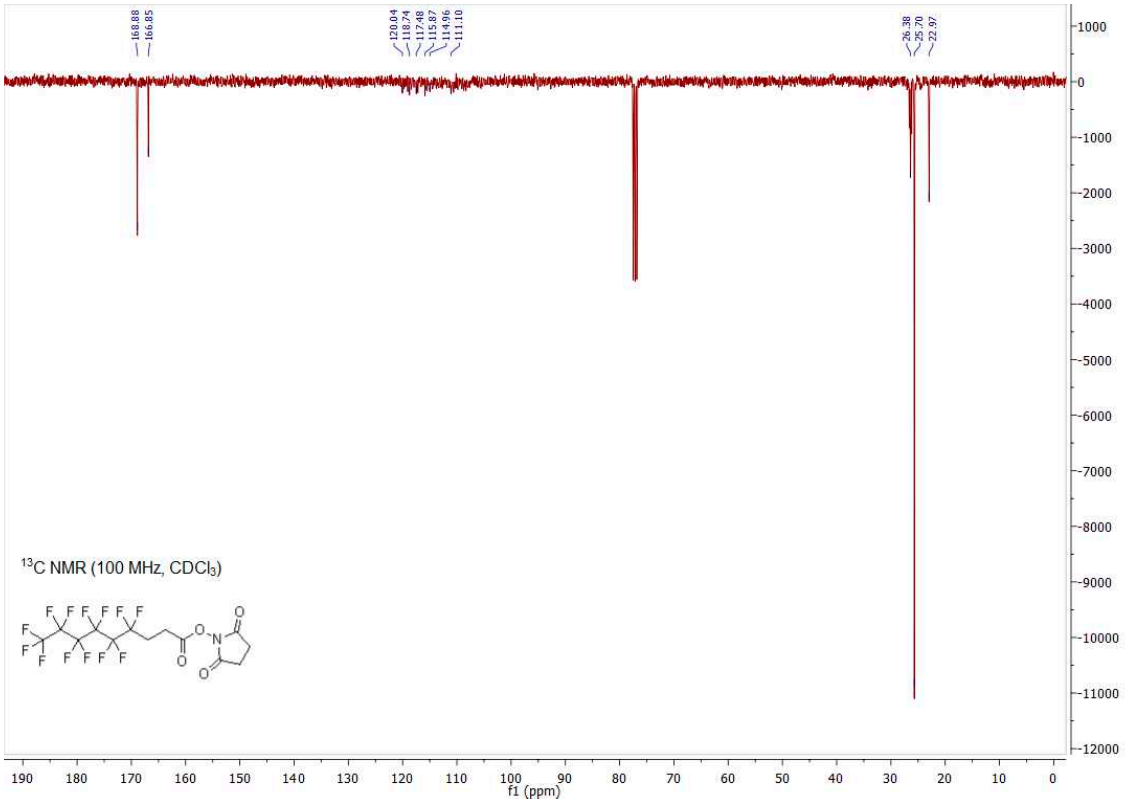

168.88
166.85
120.04
118.74
117.48
115.87
114.96
111.10
26.38
25.70
22.97
$^{13}$C NMR (100 MHz, CDCl$_3$)
f1 (ppm)

## References

Bowers, K. J., Chow, D. E., Xu, H., Dror, R. O., Eastwood, M. P., Gregersen, B. A., Klepeis, J. L., Kolossvary, I., Moraes, M. A., Sacerdoti, F. D., Salmon, J. K., Shan, Y., & Shaw, D. E. (2006). Scalable Algorithms for Molecular Dynamics Simulations on Commodity Clusters. *SC '06: Proceedings of the 2006 ACM/IEEE Conference on Supercomputing*, 43–43. https://doi.org/10.1109/SC.2006.54

Brenke, R., Kozakov, D., Chuang, G.-Y., Beglov, D., Hall, D., Landon, M. R., Mattos, C., & Vajda, S. (2009). Fragment-based identification of druggable 'hot spots' of proteins using Fourier domain correlation techniques. *Bioinformatics (Oxford, England)*, *25*(5), 621–627. https://doi.org/10.1093/bioinformatics/btp036

Friesner, R. A., Banks, J. L., Murphy, R. B., Halgren, T. A., Klicic, J. J., Mainz, D. T., Repasky, M. P., Knoll, E. H., Shelley, M., Perry, J. K., Shaw, D. E., Francis, P., & Shenkin, P. S. (2004). Glide: A new approach for rapid, accurate docking and scoring. 1. Method and assessment of docking accuracy. *Journal of Medicinal Chemistry*, *47*(7), 1739–1749. https://doi.org/10.1021/jm0306430

Halgren, T. A., Murphy, R. B., Friesner, R. A., Beard, H. S., Frye, L. L., Pollard, W. T., & Banks, J. L. (2004). Glide: A New Approach for Rapid, Accurate Docking and Scoring. 2. Enrichment Factors in Database Screening. *Journal of Medicinal Chemistry*, *47*(7), 1750–1759. https://doi.org/10.1021/jm030644s

Hoover, W. G. (1985). Canonical dynamics: Equilibrium phase-space distributions. *Physical Review A*, *31*(3), 1695–1697. https://doi.org/10.1103/PhysRevA.31.1695

Jacobson, M. P., Pincus, D. L., Rapp, C. S., Day, T. J. F., Honig, B., Shaw, D. E., & Friesner, R. A. (2004). A hierarchical approach to all-atom protein loop prediction. *Proteins*, *55*(2), 351–367. https://doi.org/10.1002/prot.10613

Johnston, R. C., Yao, K., Kaplan, Z., Chelliah, M., Leswing, K., Seekins, S., Watts, S., Calkins, D., Chief Elk, J., Jerome, S. V., Repasky, M. P., & Shelley, J. C. (2023). Epik: pKa and Protonation State Prediction through Machine Learning. *Journal of Chemical Theory and Computation*, *19*(8), 2380–2388. https://doi.org/10.1021/acs.jctc.3c00044

Jorgensen, W. L., Chandrasekhar, J., Madura, J. D., Impey, R. W., & Klein, M. L. (1983). Comparison of simple potential functions for simulating liquid water. *The Journal of Chemical Physics*, *79*(2), 926–935. https://doi.org/10.1063/1.445869

Li, J., Abel, R., Zhu, K., Cao, Y., Zhao, S., & Friesner, R. A. (2011). The VSGB 2.0 model: A next generation energy model for high resolution protein structure modeling. *Proteins*, *79*(10), 2794–2812. https://doi.org/10.1002/prot.23106

Lu, C., Wu, C., Ghoreishi, D., Chen, W., Wang, L., Damm, W., Ross, G. A., Dahlgren, M. K., Russell, E., Von Bargen, C. D., Abel, R., Friesner, R. A., & Harder, E. D. (2021). OPLS4: Improving Force Field Accuracy on Challenging Regimes of Chemical Space. *Journal of Chemical Theory and Computation*, *17*(7), 4291–4300. https://doi.org/10.1021/acs.jctc.1c00302

Martyna, G. J., Tobias, D. J., & Klein, M. L. (1994). Constant pressure molecular dynamics algorithms. *The Journal of Chemical Physics*, *101*(5), 4177–4189. https://doi.org/10.1063/1.467468

Mohamadi, F., Richards, N. G. J., Guida, W. C., Liskamp, R., Lipton, M., Caufield, C., Chang, G., Hendrickson, T., & Still, W. C. (1990). Macromodel—An integrated software system for modeling organic and bioorganic molecules using molecular mechanics. *Journal of Computational Chemistry*, *11*(4), 440–467. https://doi.org/10.1002/jcc.540110405

Ngan, C. H., Bohnuud, T., Mottarella, S. E., Beglov, D., Villar, E. A., Hall, D. R., Kozakov, D., & Vajda, S. (2012). FTMAP: Extended protein mapping with user-selected probe molecules. *Nucleic Acids Research*, *40*(Web Server issue), W271–W275. https://doi.org/10.1093/nar/gks441

Sastry, G. M., Adzhigirey, M., Day, T., Annabhimoju, R., & Sherman, W. (2013). Protein and ligand preparation: Parameters, protocols, and influence on virtual screening enrichments. *Journal of Computer-Aided Molecular Design*, *27*(3), 221–234. https://doi.org/10.1007/s10822-013-9644-8

Sherman, W., Day, T., Jacobson, M. P., Friesner, R. A., & Farid, R. (2006). Novel procedure for modeling ligand/receptor induced fit effects. *Journal of Medicinal Chemistry*, *49*(2), 534–553. https://doi.org/10.1021/jm050540c
